# TRMT1-mediated tRNA m^2^_2_G modification drives Osimertinib resistance via the ATXN3/USP25 axis in lung adenocarcinoma

**DOI:** 10.1038/s41419-026-09039-8

**Published:** 2026-06-29

**Authors:** Jiaqi Li, Bo Jing, Yingying Wang, Wenhui Bai, Qiuyu Wei, Chujiao Zhu, Xuxinyi Ling, Meng Liu, Kuofang Huang, Shifei Pan, Huishu Dong, Haijiao Lu, Jialin Qian, Yanjie Ji, Wanting Liu, Hua Zhong, Youping Zhang, Wenxuan Wu, Yuanhui Zhai, Hao Luo, Hu Lei, Hanzhang Xu, Yingli Wu, Tianqing Chu

**Affiliations:** 1 Department of Respiratory and Critical Care Medicine, Shanghai Chest Hospital, Shanghai Jiao Tong University School of Medicine, Shanghai, China; 2 Shanghai Key Laboratory of Thoracic Tumor Biotherapy, Shanghai, China; 3 Department of Clinical Laboratory Medicine, Shanghai Pulmonary Hospital, Tongji University School of Medicine, Shanghai, China; 4 Hongqiao International Institute of Medicine, Shanghai Tongren Hospital/Faculty of Basic Medicine, Chemical Biology Division of Shanghai Universities E-Institutes, Key Laboratory of Cell Differentiation and Apoptosis of the Chinese Ministry of Education, Shanghai Jiao Tong University School of Medicine, Shanghai, China; 5 The First Affiliated Hospital of Xian Jiaotong University, Xian, China; 6 Central Laboratory of Shanghai Chest Hospital, Shanghai Jiao Tong University School of Medicine, Shanghai, China

**Keywords:** Cancer therapeutic resistance, DNA methylation

## Abstract

Acquired resistance to Osimertinib, a third-generation EGFR tyrosine kinase inhibitor (TKI), remains a critical challenge in lung adenocarcinoma. Here, we identify ATXN3, a deubiquitinating enzyme, as a driver of Osimertinib resistance and a predictor of poor patient survival. Osimertinib treatment dynamically upregulates ATXN3 transcription, which stabilizes USP25 through deubiquitination, activating an ATXN3-USP25-TRMT1 signaling cascade. This axis enhances TRMT1-mediated tRNA m²_2_G modifications, enabling selective translation of redox-regulating enzymes (e.g., GPX4, SOD2) that scavenge reactive oxygen species (ROS) and mitigate drug-induced oxidative stress. Genetic ablation of TRMT1 or pharmacological targeting of USP25 with the small-molecule inhibitor AZ1 disrupted this pathway, restored ROS accumulation, and re-sensitized resistant tumors to Osimertinib in patient-derived organoids and in vivo models. Our findings reveal tRNA epitranscriptomic reprogramming as a novel mechanism of EGFR-TKI resistance and position the ATXN3/USP25/TRMT1 axis as a therapeutically actionable target to overcome Osimertinib resistance in lung cancer.

## Introduction

Epidermal growth factor receptor (EGFR) mutations drive tumorigenesis in a majority of non-small cell lung cancers (NSCLCs). Osimertinib, a third-generation EGFR tyrosine kinase inhibitor (EGFR-TKI), has transformed outcomes for advanced NSCLC patients harboring EGFR-activating mutations (e.g., T790M), yet acquired resistance inevitably develops, rendering it ineffective in nearly all cases [1–4]. While secondary EGFR mutations (e.g., C797S) and bypass signaling (e.g., MET amplification) explain resistance in ~50% of patients, the molecular drivers in the remaining cases remain poorly defined [5–7]. This knowledge gap emphasizes the critical need to identify novel resistance mechanisms and actionable vulnerabilities to extend patient survival.

Emerging evidence implicates deubiquitinating enzymes (DUBs)—key regulators of protein stability and signaling−in cancer progression and therapy resistance [8–10]. The Josephin-family DUB ATXN3 exemplifies this duality, acting as an oncogenic stabilizer of KLF4 and YAP in breast and prostate cancers while suppressing colon adenocarcinoma via Galectin-9 [11–13]. Despite the therapeutic appeal of targeting DUBs to reverse drug resistance [14, 15], their role in Osimertinib-refractory NSCLC remains unexplored.

Beyond genomic alterations, post-transcriptional reprogramming, including transfer RNA (tRNA) modifications, is increasingly linked to therapy evasion. Chemical modifications (e.g., m^7^G, m^2^_2_G) fine-tune tRNA stability and translational fidelity, with dysregulation contributing to malignancies and treatment failure [16–18]. For example, METTL1-mediated tRNA m^7^G modification drives lenvatinib resistance in hepatocellular carcinoma [19], yet whether tRNA epitranscriptomics underpins EGFR-TKI resistance remains unknown.

Here, we resolve these gaps by identifying a DUB-driven tRNA modification axis that sustains Osimertinib resistance. We demonstrate that adaptive upregulation of *ATXN3* stabilizes the DUB USP25, which in turn enhances tRNA methyltransferase TRMT1 to boost m^2^_2_G modifications. This rewires redox homeostasis via selective translation of GPX4 and SOD2, enabling tumor cells to evade Osimertinib-induced oxidative stress. Targeting this axis with the clinical-stage USP25 inhibitor AZ1 restored Osimertinib sensitivity in patient-derived organoids and murine models, nominating ATXN3/USP25/TRMT1 as a druggable nexus to combat resistance. Our work bridges DUB activity, tRNA epitranscriptomics, and redox adaptation, offering a translatable strategy to prolong EGFR-TKI efficacy.

## Materials and methods

### Cells and cell culture

HCC827, HCC827OR (Osimertinib-resistant), H1975, and H1975OR (Osimertinib-resistant) cell lines were provided by the Department of Pharmacology, Shanghai Jiao Tong University School of Medicine. We maintained and validated the resistant cell lines with Osimertinib (Fig. S1). Detailed information on these cell lines has been described in previously published articles [20]. All cell lines used in this study were authenticated by short-tandem-repeat (STR) profiling.

### Quantitative RT-PCR

Total RNA was extracted using TRIzol (TaKaRa, Shiga, Japan), quantified (NanoDrop 2000, Thermo, Waltham, MA, USA), and reverse-transcribed with PrimeScript RT Master Mix (TaKaRa). Q-PCR was performed using TB Green Premix (TaKaRa) on a QuantStudio 6 Pro system (Applied Biosystems, Foster City, CA, USA) with primers listed in Table S1. Gene expression was normalized to GAPDH via the ΔΔCt method.

### Immunoblotting

Cells were lysed in RIPA buffer (Beyotime, Shanghai, China) with protease/phosphatase inhibitors (Roche, Mannheim, Germany). Proteins (20 μg/lane) were resolved on 10% SDS-PAGE gels, transferred to PVDF membranes (Millipore, Burlington, MA, USA), blocked with 5% non-fat milk, and probed overnight at 4 °C with primary antibodies: ATXN3 (1:1000, # 13505-1-AP, Proteintech, Rosemont, IL, USA), USP25 (1:1000, #12199-1-AP, Proteintech), TRMT1 (1:1000, #14970-1-AP, Proteintech), β-actin (1:5000, #A5441, Sigma, St. Louis, MO, USA). α-Tubulin (1:1000, #9099, Cell Signaling, Danvers, MA, USA). Blots were developed with HRP-conjugated secondary antibodies (1:5000, #7074, #7076, Cell Signaling) and SuperSignal West Pico ECL (Thermo), visualized using a ChemiDoc MP system (Bio-Rad, California, USA).

### Data acquisition and analysis

RNA sequencing data (FPKM/TPM normalized), clinical metadata, and survival outcomes for lung adenocarcinoma (LUAD) patients were obtained from The Cancer Genome Atlas (TCGA-LUAD cohort, dbGaP#9112) alongside normal lung tissue expression profiles from the Genotype-Tissue Expression project (GTEx v8). Batch effects between datasets were adjusted using ComBat (sva R package). Raw counts were normalized via DESeq2 for differential expression analysis (FDR < 0.05), while survival analysis employed Kaplan-Meier curves. All analyses were performed in R (v4.1.2).

### CRISPR-Cas9-mediated gene depletion

Single-guide RNAs (sgRNAs) targeting ATXN3, USP25, and TRMT1 were designed using CRISPOR (https://crispor.gi.ucsc.edu/), synthesized as oligonucleotides, and cloned into the BbsI site of the pSpCas9(BB)-2A-Puro (PX459) V2.0 vector (#62988, Addgene, Watertown, MA, USA) via T4 DNA ligase. sgRNA sequences included: sgATXN3 #4 (TGCCTGAATAACTTATTGCA), sgATXN3 #6 (CTGTCATCCATATTTCCAGA), sgUSP25 #3 (CCCGTTGGGCTAAAGAATGT), sgUSP25 #4 (CTGATGTATCTATCATTGCC), sgTRMT1 #2 (AAGCTGAATGCGAGCAAACT), and sgTRMT1 #3 (GAGGTTTGACGTCATCGATC). Constructs were transfected into target cells using Lipofectamine 3000, followed by puromycin selection (1–2 µg/mL) to generate polyclonal populations. Depletion efficiency was validated by Sanger sequencing and Western Blot.

### PLVX plasmid-mediated gene overexpression

The coding sequences (CDSs) of ATXN3, USP25, and TRMT1 were obtained from commercially available human cDNA ORF clones (ATXN3 clone: #Z7561, NM_004993.6; USP25 clone:#E3475 NM_001283041.3; TRMT1 clone: #V0400, NM_001136035.4; GeneCopoeia, Rockville, MD USA) and PCR-amplified from cDNA using primers flanked by restriction sites, EcoRI/XhoI, cloned into the PLVX-Puro vector (#180635, Addgene), and sequence-verified by Sanger sequencing. Lentiviral particles generated via HEK293T cell transfection were used to transduce target cells, followed by puromycin selection (1–2 µg/mL) to establish stable lines. Overexpression was confirmed via Western Blot by detecting tags anti-Flag(#66008-4-Ig, Proteintech) or anti-Myc(#60003-2-Ig, Proteintech).

### Cell growth and viability assay

Cells were seeded into 96-well plates at 4000 cells/well and maintained under standard culture conditions (37 °C, 5% CO_2_). Cell proliferation was quantified using the Cell Counting Kit-8 (CCK-8, #CK04-11, Dojindo Molecular Technologies, Munich, Germany) according to the manufacturer’s protocol. At designated time points, 10 µL of CCK-8 reagent was added to each well, followed by incubation for 1–4 h. Absorbance was measured at 450 nm using a microplate reader (BioTek Synergy H1, Winooski, VT, USA).

### Lentiviral production and transduction

Lentiviruses were generated by co-transfecting HEK293T cells with the transfer plasmid, psPAX2 (#12260, Addgene), and pMD2G (#12259, Addgene) using a transfection reagent (Lipofectamine 3000, Thermo Fisher). Viral supernatants were harvested 48 h post-transfection, filtered (0.45 µm), and used to transduce HCC827 and H1975 cells in the presence of polybrene (6 µg/mL; MCE, Monmouth Junction, NJ, USA). After 24 h, transduced cells were selected with puromycin (1 µg/mL; Beyotime). Successful knockdown or overexpression was confirmed by Western Blot.

### Colony formation assay

Cells were seeded into 12-well plates at a density of 800 cells per well and cultured in complete medium for ~14 days. Colonies were fixed with 4% paraformaldehyde (15 min), stained with 0.01% crystal violet (10–15 min), and gently washed to remove excess dye. Colonies containing >50 cells were quantified using ImageJ software (National Institutes of Health) with size/threshold adjustments for consistency.

### Cell apoptosis assay

Cell apoptosis was assessed using the Annexin V-FITC/PI Apoptosis Detection Kit (#C1062M, Beyotime) per the manufacturer’s protocol. Briefly, cells were dual-stained with Annexin V-fluorescein isothiocyanate (FITC) and propidium iodide (PI), followed by flow cytometry analysis (BD FACSCanto™ II) to distinguish viable (FITC − /PI − ), early apoptotic (FITC + /PI − ), late apoptotic (FITC + /PI + ), and necrotic (FITC − /PI + ) populations.

### Co-immunoprecipitation (Co-IP) and silver staining

H1975OR cells (5 × 10^7^) were lysed in 1 mL of lysis buffer(50 mM Tris, pH 7.4; 150 mM NaCl; 1 mM EDTA; 1% Triton X-100; 10% Glycerol, Protease inhibitors) supplemented with protease inhibitors. Lysates were incubated overnight at 4 °C with primary antibodies or normal IgG (control, #68860, #2729, Cell Signaling) alongside Protein A/G Plus agarose beads (#sc-2003, Santa Cruz Biotechnology, Dallas, TX, USA). Beads were washed three times with washing buffer(50 mM Tris, pH 7.4; 150 mM NaCl; 1 mM EDTA; 1% Triton X-100; 10% Glycerol), and bound proteins were eluted in 1×SDS loading buffer. Eluates were resolved by SDS-PAGE and silver-stained using a Fast Silver Staining Kit (Beyotime). Distinct protein bands were excised, processed for LC-MS/MS identification, and co-immunoprecipitated proteins were further validated by Western Blot.

### RNA-seq analysis

Total RNA was extracted from samples using the Universal RNA Extraction CZ Kit (#RNC643, ONREW, Foshan, China) according to the manufacturer’s instructions. RNA libraries were prepared with the VVAHTS® Universal V8 RNA-seq Library Prep Kit for Illumina (#NR605-0, Vazyme, Nanjing, China) and sequenced on an Illumina NovaSeq 6000 platform (150 bp paired-end reads). Raw reads were processed via Skewer v0.2.2, and clean reads were aligned to the human reference genome (GRCh38, Ensembl) with STAR, allowing one mismatch. Gene expression quantification was performed using StringTie v1.3.1c, and differential expression analysis ( | fold change | ≥ 2, *P* < 0.05) was conducted via DESeq2 v1.16.1. Differentially expressed genes (DEGs) underwent functional enrichment and pathway analysis using the TopGO database, with significance thresholds set at *P* < 0.05.

### Immunofluorescence

Cells were fixed with 4% paraformaldehyde (PFA) and permeabilized with 0.5% Triton X-100 in PBS, followed by blocking in 2% bovine serum albumin (BSA) in PBS for 1 h at room temperature. Samples were incubated with primary antibodies and subsequently with Alexa Fluor 488- or 594-conjugated secondary antibodies (Invitrogen, Carlsbad, CA, USA), with nuclei counterstained using 4,6-diamidino-2-phenylindole (DAPI). Confocal images were acquired on a Nikon laser confocal microscope.

### Measurement of reactive oxygen species (ROS)

ROS levels in cells were quantified using 2’,7’-dichlorodihydrofluorescein diacetate (DCFH-DA, #D6883, Sigma-Aldrich). DCFH-DA was diluted in serum-free cell culture medium to a final concentration of 10 µM (1:1000 dilution) and protected from light. Cells were washed twice with PBS (#10010023, Gibco, Grand Island, NY, USA), incubated with DCFH-DA for 20 min at 37 °C/5% CO₂, and washed three times with serum-free TSM to remove excess probe. Cells were dissociated using TrypLE™ (Thermo Fisher, #12605010), filtered through a 40 µm strainer (#431750, Sigma), and resuspended in PBS containing 2% FBS (#10437028, Gibco). Fluorescence intensity (ex/em: 488/525 nm) was measured using a BD FACSAria™ III flow cytometer (BD Biosciences, San Jose, CA, USA) with FACSDiva™ software (v8.0.1). Untreated cells and unstained controls (medium-only + DCFH-DA) served as negative controls, while 100 µM H₂O₂ (#H1009, Sigma-Aldrich)-treated cells were used as a positive control. Data were analyzed using FlowJo™ (v10.8.1, BD Biosciences), with ROS levels expressed as median fluorescence intensity (MFI) fold change relative to untreated controls. Statistical significance was determined via one-way ANOVA with Tukey’s post hoc test (*p* < 0.05) in GraphPad Prism (v9.0).

### Measurement of redox enzyme activity

GPX activity was measured using a Glutathione Peroxidase (GPX) Activity Assay Kit (# S0056, Beyotime) according to the manufacturer’s instructions. SOD activity was determined using a Total SOD Activity Assay Kit (NBT method, #S0109, Beyotime), based on inhibition of nitroblue tetrazolium (NBT) reduction. Catalase activity was measured using a Catalase Activity Assay Kit (#S0051, Beyotime), which quantifies hydrogen peroxide decomposition. Enzyme activities were normalized to total protein concentration.

### Polysome profiling and quantitative PCR (Q-PCR)

Polysome profiling was performed as previously described [21] with modifications. Briefly, cell pellets (5 × 10⁶ cells/sample) were resuspended in ice-cold PBS (#10010023, Gibco) and lysed in polysome lysis buffer (10 mM HEPES-KOH pH 7.4, 100 mM KCl, 5 mM MgCl₂, 1% Triton X-100, 100 µg/mL cycloheximide) supplemented with RNase inhibitor (#2313B, Takara). Lysates were clarified by centrifugation (12000 × *g*, 10 min, 4 °C) and layered onto 10–50% linear sucrose gradients (#S7903, Sigma-Aldrich) prepared in SW41 Ti ultracentrifuge tubes (#344059, Beckman Coulter, Brea, CA, USA). Gradients were centrifuged at 35000 rpm for 2.5 h at 4 °C (Optima XE-90 ultracentrifuge, Beckman Coulter) and fractionated using a Brandel BR-188 Density Gradient Fractionation System with continuous A₂₅₄ nm monitoring (UA-6 UV detector, Teledyne ISCO). Fractions corresponding to monosomes (40S/60S), light polysomes (2–4 ribosomes), and heavy polysomes ( > 5 ribosomes) were collected based on elution profiles. RNA was extracted from pooled fractions using TRIzol™ LS (#10296028, Thermo Fisher), reverse-transcribed with SuperScript IV (#18091050, Thermo Fisher), and analyzed by Q-PCR using PowerUp™ SYBR Green Master Mix (#A25742, Thermo Fisher) on a QuantStudio 6 Flex thermocycler (Applied Biosystems). Primer pairs (10 µM; Integrated DNA Technologies) for target mRNAs (*GPX4*, *SOD2, Catalase*) and housekeeping controls (*GAPDH*) were validated for efficiency (90–110%). Data were normalized to *GAPDH* and analyzed via the ΔΔCt method using QuantStudio Software v1.7.1.

### LC-MS-based tRNA modification profiling

tRNA was isolated from total RNA extracted from cells (1 × 10⁷ cells/sample; TRIzol™, #15596026, Thermo Fisher) using denaturing urea-polyacrylamide gel electrophoresis (#4566053, Bio-Rad). The 60–90 nt tRNA band was excised under UV shadowing (254 nm) alongside an RNA marker (Decade™ Marker, #AM7778, Thermo Fisher), passively eluted overnight in 0.3 M NaCl, and precipitated with ethanol (2.5 volumes) and glycogen carrier (5 µg/mL, #AM9510, Thermo Fisher). Purified tRNA (1 µg/µL) was enzymatically digested to nucleosides using nuclease P1 (0.5 U/µg, #N8630, Sigma-Aldrich) in 20 mM ammonium acetate (pH 5.3, 2 h, 37 °C), followed by dephosphorylation with antarctic phosphatase (#B0289S, NEB, Ipswich, MA, USA) and phosphodiesterase I (0.002 U/µL, #P3243, Sigma-Aldrich) in 10 mM Tris-HCl (pH 7.5, 1 h, 37 °C). Deproteinization was performed using a 10 kDa MWCO spin filter (Vivacon® 500, #VN01H01, Sartorius, Göttingen, Germany) at 14000 × *g* (15 min, 4 °C). Nucleosides were separated on an Agilent 1290 HPLC system equipped with a Zorbax Eclipse Plus C18 column (2.1 × 50 mm, 1.8 µm; #959757-902, Agilent, Santa Clara, CA, USA) at 40 °C. Mobile phases: (A) 0.1% formic acid; (B) acetonitrile/0.1% formic acid. Gradient: 0–5 min, 0–5% B; 5–20 min, 5–95% B (flow rate: 0.3 mL/min). Detection used an Agilent 6460 QQQ mass spectrometer in positive ESI mode (capillary voltage: 3500 V; gas temp: 300 °C; nebulizer: 35 psi). Modified nucleosides (e.g., m¹A, Ψ, m⁷G) were quantified via MRM transitions (e.g., m¹A: 282.1 → 150.1, CE 20 V) against a synthetic standard mix, with a matrix blank (no tRNA) and E. coli tRNA (#R4251, Sigma-Aldrich) as controls. Data were acquired using Agilent MassHunter (v10.0), normalized to unmodified guanosine (ΔA₂₆₀), and analyzed via Student’s *t*-test (*p* < 0.05) in Prism 9.0.

### tRNA sequencing (tRNA-seq) analysis

Purified tRNA (1 µg/sample; TRIzol™, Thermo Fisher, #15596026) underwent sequential demethylation: rtStar^TM^ tRF&tiRNA Pretreatment Kit (AS-FS-005, Arraystar, Rockville, MD, USA) was used for tRNA m1A&m3C demethylation treatment. 50 µL demethylation reaction mixture was prepared according to the manufacturer’s protocol and incubated at 37 °C for 2 hours. Then 40 µL nuclease-free water and 10 µL 5×Stop Buffer were added to terminate the reaction. Demethylated tRNA was purified by phenol-chloroform extraction and ethanol precipitation.

Demethylated tRNA was partially hydrolyzed in 50 mM Na₂CO₃/NaHCO₃ (pH 9.4, 90°C, 20 min), dephosphorylated with CIP (1 U/µg; NEB, #M0290S) in CutSmart Buffer (30 min, 37 °C), and re-phosphorylated with T4 PNK (10 U/µg; #M0201S, NEB) in 1×T4 PNK Buffer (1 mM ATP, 30 min, 37 °C). Fragments (19-35 nt) were size-selected on a 15% TBE-urea gel (Bio-Rad, #4566053) and converted to Illumina-compatible libraries using the NEBNext® Multiplex Small RNA Library Prep Kit (#E7300S, NEB).

Libraries were quantified via Qubit™ 4.0 (#Q33238, Thermo Fisher) and qualified with an Agilent BioAnalyzer 2100 (High Sensitivity DNA Kit, #5067-4626, Agilent). Sequencing was performed on an Illumina NextSeq 500 (75-cycle High Output Kit, #20024906, Illumina, San Diego, CA) with 50 bp single-read configuration. Raw reads were adapter-trimmed (Cutadapt v3.5), mapped to hg38 tRNA annotations (GtRNAdb) using Bowtie2 (v2.4.5), and quantified with tRNAExplorer(https://github.com/hqyone/tRNAExplorer/blob/master/help/tRNAExplorer_manual.md) [22]. Differential expression analysis was performed using DESeq2 (v1.38.3). Raw read counts were normalized using spike-in controls (#339390, Qiagen, Germantown, MD, USA) to account for technical variation across samples. RNAs with an adjusted false discovery rate (FDR) < 0.05 and an absolute log2 fold change ( | log2FC | ) > 1 were considered significantly differentially expressed.

### Calculation method for expression‑weighted tRNA decoding weight

We estimated an expression‑weighted tRNA decoding contribution for each gene by combining codon demand from the gene CDS and tRNA supply measured in 1975OR cells by tRNA‑seq. Specifically, MANE Select CDSs of GPX4, SOD2, and CAT were parsed into in‑frame codons, and codon counts were obtained. For each codon, the set of tRNAs annotated to decode it was taken from the tRNA annotation, and each tRNA was weighted by its abundance in 1975OR cells (from the tRNAseq Expression_Count table). For a given codon, the relative contribution of each decoding tRNA was proportional to its abundance among all tRNAs capable of decoding that codon. The gene‑level decoding weight of each tRNA was then calculated as the sum of its per‑codon contributions across the CDS, weighted by codon counts and normalized by the total number of CDS codons. tRNAs were ranked by this weight to report and visualize the top contributors for each gene.

### Synergy analysis

Synergy scores were calculated using SynergyFinder 3.0 (https://synergyfinder.fimm.fi) with the ZIP model [23]. Dose-response matrices (*n* = 3 b) were uploaded, and synergy scores were classified as: synergistic (ZIP synergy score > 10), additive (0 ≤ ZIP synergy score ≤ 10), or antagonistic (ZIP synergy score < 0).

### Tumor xenograft studies

All animal procedures were approved by the Institutional Animal Care and Use Committee and conducted in accordance with ARRIVE guidelines. Mycoplasma-free, STR-authenticated HCC827OR parental, USP25-KO, and TRMT1-KO cells were harvested at 80% confluence using TrypLE™ (#12605010, Thermo Fisher), washed with ice-cold PBS (#10010023, Gibco), and resuspended in PBS at 1 × 10⁷ cells/200 µL (viability >95% via Trypan Blue exclusion). Female BALB/c nude mice (6–8 weeks) were subcutaneously injected with 200 µL cell suspension (*n* = 5) in the right axilla and housed under SPF conditions (12 h light/dark, 22 °C, 50% humidity). Mice were randomly allocated to experimental groups. Tumor volume was measured every 48 h using digital calipers and calculated as V = L×W^2^/2, where L and W represent the longest axis and perpendicular width, respectively. Mice were euthanized by CO₂ asphyxiation upon reaching a tumor volume of 1500 mm³ or at day 28 post-implantation; excised tumors were weighed, snap-frozen in liquid nitrogen, and stored at –80 °C. Tumor measurements were not performed blinded; however, calipers and the final tumor weighing followed identical objective protocols across groups.

### Histology and immunohistochemistry (IHC) and progression-free survival (PFS) analysis

Human tumor tissues were obtained from patients with advanced lung adenocarcinoma under protocols approved by the Ethics Committee of Shanghai Chest Hospital (Approval #KS24041. Inclusion Criteria: 1: Lung adenocarcinoma patients receiving Osimertinib or other EGFR-TKIs treatment; 2: Obtained tissue biopsy before and after Osimertinib or other EGFR-TKIs treatment; 3: Follow-up information obtained. Exclusion Criteria: 1: The quality of the obtained tissue biopsy is poor; 2: Patients lost to follow-up. Tissues fixed in 4% formaldehyde (#033314-K2, Thermo Fisher), paraffin-embedded, and sectioned (4 µm). For IHC, sections were deparaffinized, subjected to antigen retrieval in citrate buffer (pH 6.0; #C9999, Sigma-Aldrich), blocked with 5% BSA (#A7030-50G, Sigma-Aldrich), and incubated overnight at 4 °C with primary antibodies (anti-USP25; Proteintech, 12199-1-AP, 1:200; anti-TRMT1; Proteintech, 14970-1-AP, 1:200), followed by HRP-conjugated secondary antibodies and DAPI counterstaining (#D1306, Thermo Fisher). Double IHC used sequential staining with chromogenic substrates (DAB/Vector Blue, #SK-4105, #SK-5300, SZABO-SCANDIC, Vienna, Austria). Negative (primary antibody omitted) and positive controls validated specificity. Staining intensity was quantified using Fiji [24]. Response Evaluation Criteria in Solid Tumors (RECIST) v1.1 was used to evaluate tumor response. PFS is defined as the time from initiation of Osimertinib to disease progression or death.

### Culture of patient-derived tumor organoid models

Tumor tissues from three patients with advanced lung adenocarcinoma were obtained under protocols approved by the IACUC of Shanghai Lide. Patient 1 (EGFR 19del, post-Osimertinib resistance with T790M loss), Patient 2 (EGFR L858R/BRAF + , post-Osimertinib/c-Met amplification), and Patient 3 (EGFR 19del, post-Osimertinib resistance without known resistance mutations) were subcutaneously implanted into 6-week-old NSG mice (Jackson Laboratory, #005557). Tumors ( ≈ 500 mm³) were dissected into 1–3 mm³ fragments, digested in Collagenase/Hyaluronidase buffer (StemCell Technologies, #07912, 37 °C, 1–2 h), filtered (70 µm, #431751, Corning), centrifuged (300 × *g*, 3 min), and resuspended in tumor sphere medium (#1701, ScienCell) supplemented with 12 µg/mL collagen I (#354236, Corning). Cells (1.13 × 10⁵/mL) were seeded into ultra-low-attachment plates (#3471, Corning), centrifuged (100 × *g*, 3 min), and cultured at 37 °C/5% CO₂ with half-medium changes every 3 days. Organoid viability was confirmed via Calcein-AM/propidium iodide staining (#C3099/P3566, Thermo Fisher), and mycoplasma testing (#LT07-318, Lonza) was performed monthly.

### PDXOs drug testing

Drug testing was performed under protocols approved by the Institutional Review Board (IRB) and the Institutional Animal Care and Use Committee (IACUC) of Shanghai Lide. High-concentration drug stocks were diluted in tumor sphere medium (TSM, #1701, ScienCell) to 20×working solutions containing 2% DMSO (#D2650, Sigma-Aldrich). For treatment, 135 µL of PDXO cell suspension (1.13 × 10⁵ cells/mL) was combined with 7.5 µL of 20×drug or TSM control in 96-well ultra-low-attachment plates (#3471, Corning), achieving a final DMSO concentration of 0.2% (total volume: 150 µL/well). CellTiter-Glo® 3D reagent (50 µL/well, #G9683, Promega) was added on days 0, 2, 4, and 6 of post-treatment. Plates were incubated for 10 min at room temperature, and luminescence (ATP content) was measured (80 µL/well) using a SpectraMax® i3x multi-mode reader (Molecular Devices). Inhibition rates were calculated as:

Inhibition rate (%) = ((V_control_-V_blank_)-(V_drug_-V_blank_))/(V_control_-V_blank_)×100%. Where V_control_, V_drug_, and V_blank_ represent untreated, drug-treated, and medium-only wells, respectively. Data were analyzed using GraphPad Prism v9.0 (two-way ANOVA, *p* < 0.05).

### Sample-size estimation

No formal a priori statistical power analysis was performed.

Sample sizes for in-vitro experiments (*n* = 3 independent biological replicates per condition) and for tumor-xenograft experiments (*n* = 5 mice per group, see “Tumor Xenograft Studies”) were chosen based on standards in the field and our prior work using the same isogenic Osimertinib-sensitive/resistant LUAD cell pairs [20]. For human IHC and PFS analyses, all eligible paired pre-/post-treatment biopsies meeting the inclusion criteria during the recruitment window (Approval #KS24041) were included.

### Statistical analysis

Data are presented as means ± standard error of the mean (SEM) from at least three independent biological replicate experiments, analyzed using GraphPad Prism software unless otherwise indicated in the figure legend. When error bars are not visible, they are within the symbol area. Statistical analysis mainly employed the two-tailed Student’s *t* test or analysis of variance (ANOVA) with Tukey’s multiple-comparison test, unless otherwise specified.

Normality and homogeneity of variance were assessed by Shapiro–Wilk/Brown–Forsythe/visual inspection. When assumptions were not met, the corresponding non-parametric test (Mann–Whitney U or Kruskal–Wallis with Dunn’s correction) was used. Within-group variation is reported as mean ± SEM. Multiple-comparison adjustment was Tukey’s HSD for ANOVA. Statistical significance is denoted as follows: *P < 0.05, **P < 0.01, ***P < 0.001, ****P < 0.0001.

## Results

### ATXN3 is upregulated in Osimertinib-resistant LUAD models and correlates with poor clinical outcomes

**Fig. 1:**
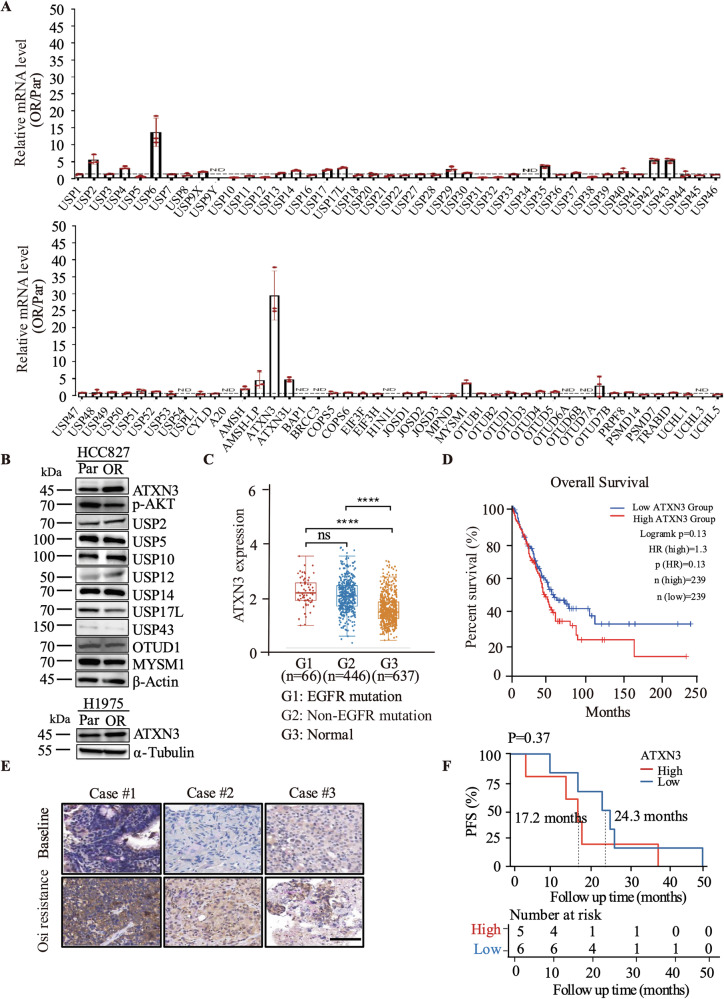
ATXN3 is upregulated in Osimertinib-resistant cells and correlates with poor prognosis. **A** Q-PCR analysis of 86 deubiquitinating enzymes (DUBs) in H1975 and H1975OR lung adenocarcinoma cells. *n* = 3; Mean ± SEM; Student’s t test. **B** Western Blot of DUB protein levels in sensitive vs. resistant HCC827 cells. **C** TCGA dataset analysis of ATXN3 in lung adenocarcinoma (LUAD) tumors. **D** Kaplan-Meier survival analysis of LUAD patients (TCGA) stratified by ATXN3 mRNA expression (high vs. low, median cutoff). **E** Representative immunohistochemistry (IHC) images of ATXN3 protein in paired pre- and post-Osimertinib resistance biopsies from three patients. Brown: ATXN3; blue: hematoxylin. Scale bar: 50 μm. **F** Progression-free survival (PFS) of patients with low baseline ATXN3 expression and high expression treated with Osimertinib (Median PFS:24.3 vs. 17.2 months, *P* = 0.37). (**C**) *p < 0.05; **p < 0.01; ***p < 0.001; ****p < 0.0001.

We performed qRT-PCR screening of 86 deubiquitinating enzymes (DUBs) in isogenic Osimertinib-sensitive (HCC827, H1975) and -resistant (HCC827OR, H1975OR) lung adenocarcinoma (LUAD) cell lines. Resistant clones exhibited 122-fold and 46-fold increases compared to sensitive clones **(**Fig. S1**)**. Among screened DUBs, *ATXN3* mRNA was most significantly elevated in resistant cells (Fig. 1A). The protein level of ATXN3 was also significantly elevated in resistant cell lines with no changes in other DUBs (Fig. 1B). Analysis of TCGA-LUAD and GTEx normal lung RNA-seq datasets revealed elevated *ATXN3* expression in lung tumors (Fig. 1C), consistent in *EGFR*-mutant and *EGFR*-WT subgroups. High *ATXN3* expression correlated with reduced median overall survival (Fig. 1D). Immunohistochemistry (IHC) of paired pre-/post-Osimertinib LUAD specimens demonstrated increased ATXN3 protein post-resistance (Fig. 1E). Then, we divided patients into low and high ATXN3 expression groups based on IHC quantification results and evaluated the correlation between baseline ATXN3 levels in patients and progression-free survival (PFS) with Osimertinib treatment. Patients with low ATXN3 expression showed a trend toward longer PFS compared with those with high ATXN3 expression (Fig. 1F).

### ATXN3 drives osimertinib resistance in lung adenocarcinoma by enhancing proliferation and survival

**Fig. 2:**
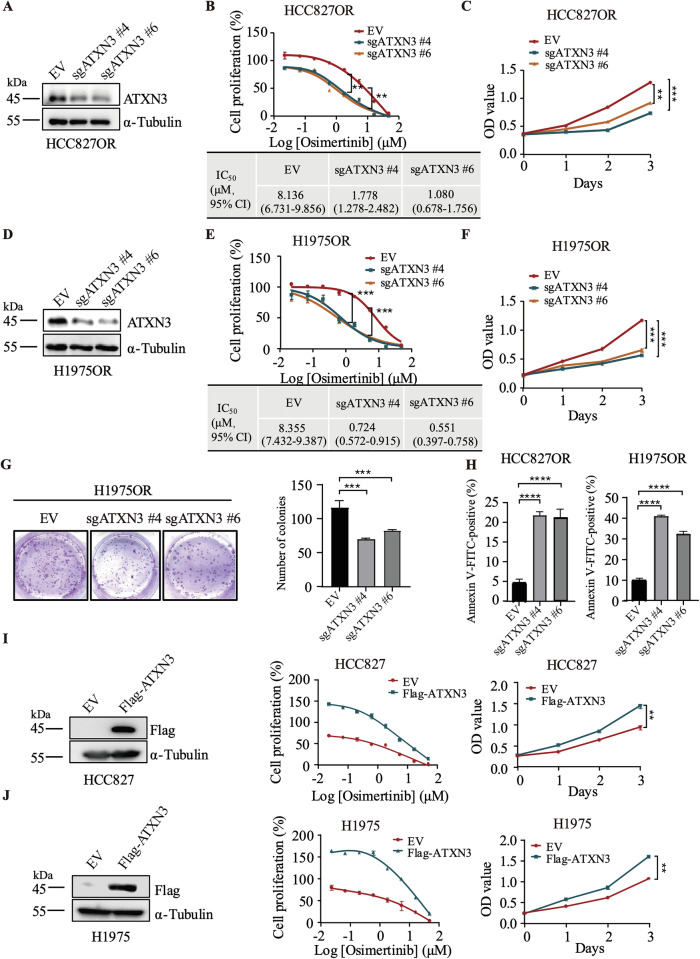
ATXN3 promotes Osimertinib resistance by enhancing proliferation and suppressing apoptosis in lung adenocarcinoma. HCC827OR and H1975OR cells were transfected with the control EV or ATXN3 sgRNAs. Cellular extracts were collected for Western Blot with indicated antibodies (**A**, **D**). The sensitivity to Osimertinib and proliferation ability were measured by CCK8 (**B**, **C**, **E**, **F**). Colony formation assay and Quantitative analysis of H1975OR cells ± ATXN3 KO cultured for 14 days (**G**). Apoptosis rate were established of HCC827OR cells ± ATXN3 KO and H1975OR cells ± ATXN3 KO treated with Osimertinib (1 μM) for 48 h by flow cytometry analysis of Annexin-V-PI dual staining(**H**). **I**, **J** Overexpression of ATXN3 in HCC827 and H1975 cells was verified by Western Blot, the sensitivity to Osimertinib and the proliferation ability were measured by CCK8. (**B**, **E**) *n* = 3; IC_50_ values were estimated using nonlinear regression and are presented with 95% confidence intervals. Differences in IC_50_ values between groups were assessed using an extra sum-of-squares F test. (**C**, **F**, **G**, **H**, **I**) *n* = 3; Mean ± SEM; Analysis of variance (ANOVA) with Tukey’s multiple-comparison test. *p < 0.05; **p < 0.01; ***p < 0.001; ****p < 0.0001. EV: Empty vector.

To further investigate the role of ATXN3 in Osimertinib resistance, we generated ATXN3-depletion HCC827OR (Fig. 2A). Treatment with escalating Osimertinib concentrations for 72 h revealed that ATXN3 deletion significantly reduced IC_50_ values to Osimertinib and proliferation compared to controls (Fig. 2B, C). The same phenomenon was also observed in ATXN3-depletion 1975OR cells(Fig. 2D–F). Consistently, clonogenic survival was severely impaired in ATXN3-deficient resistant cells (Fig. 2G). Furthermore, Annexin V/PI staining revealed an increase in apoptosis in ATXN3-KO HCC827OR and H1975OR cells (Fig. 2H). Conversely, ectopic ATXN3 overexpression in Osimertinib-sensitive HCC827 and H1975 cells enhanced drug resistance and proliferation (Fig. 2I, J). These data establish ATXN3 as a critical mediator of Osimertinib resistance, promoting survival and proliferation in lung adenocarcinoma.

### USP25 physically and functionally interacts with ATXN3 to sustain resistance

**Fig. 3:**
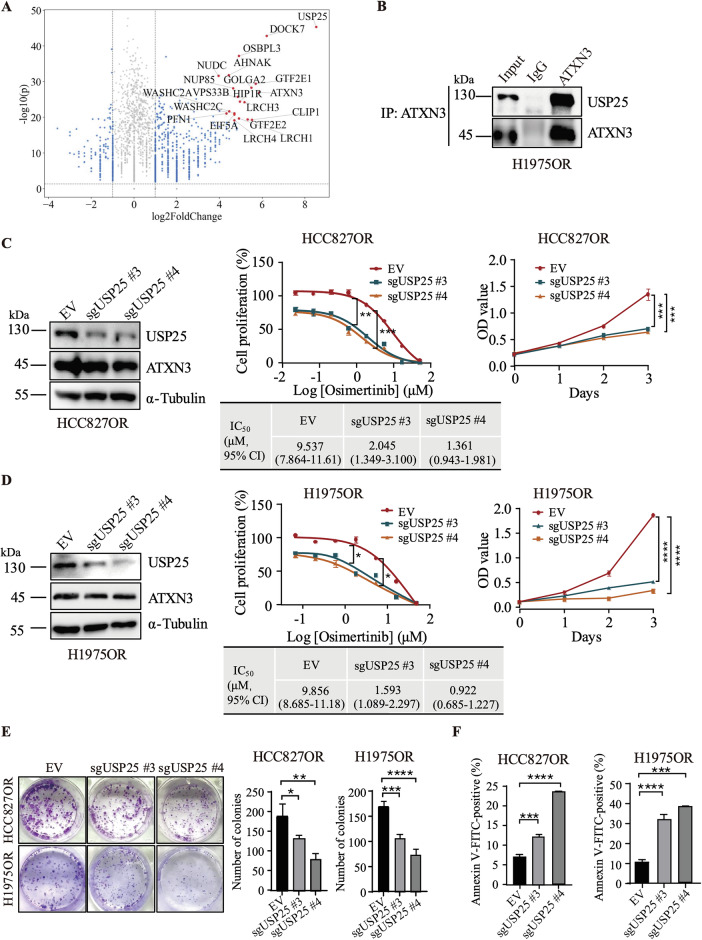
USP25 interacts with ATXN3 to promote Osimertinib resistance, proliferation, and colony formation while suppressing apoptosis. **A** Volcano plot displaying differentially expressed proteins from quantitative proteomic analysis of ATXN3. **B** Co-IP analysis of the association between ATXN3 and USP25 in H1975OR cells and lysates immunoprecipitated with antibodies against the indicated proteins. HCC827OR and H1975OR cells were transfected with the control EV or USP25 sgRNAs. Cellular extracts were collected for Western Blot with indicated antibodies and the sensitivity to Osimertinib and proliferation ability were measured by CCK8 (**C**, **D**). Colony formation assay and Quantitative analysis of H1975OR cells ± USP25 KO cultured for 14 days (**E**). Apoptosis rate were established of HCC827OR/H1975OR cells ±USP25 KO treated with Osimertinib (1 μM) for 48 h by flow cytometry analysis of Annexin-V-PI dual staining (**F**). **C**, **D**, **E** *n* = 3; Mean ± SEM; Analysis of variance (ANOVA) with Tukey’s multiple-comparison test. IC_50_ values were estimated using nonlinear regression and are presented with 95% confidence intervals. Differences in IC_50_ values between groups were assessed using an extra sum-of-squares F test. *p < 0.05; **p < 0.01; ***p < 0.001; ****p < 0.0001. EV: Empty vector.

In order to identify ATXN3-associated effectors, immunoprecipitation-mass spectrometry (IP-MS) of ATXN3 complexes in resistant cells identified USP25 as the predominant interactor (Fig. 3A). Co-immunoprecipitation (Co-IP) confirmed binding between endogenous ATXN3 and USP25 (Fig. 3B). USP25 deletion in HCC827OR and H1975OR cells recapitulated the phenotype of ATXN3 depletion, enhancing Osimertinib sensitivity, inhibiting cell growth (Fig. 3C, D), suppressing colony formation (Fig. 3E), and increasing apoptosis (Fig. 3F). Notably, USP25 depletion did not alter ATXN3 protein levels (Fig. 3D), suggesting a non-degradative partnership. Conversely, USP25 overexpression in drug-sensitive cells enhanced Osimertinib resistance and proliferation (Fig. S2). Collectively, these findings delineate an ATXN3-USP25 axis that cooperatively drives Osimertinib resistance through enhanced survival and proliferation.

### ATXN3 promotes Osimertinib resistance by stabilizing USP25

**Fig. 4:**
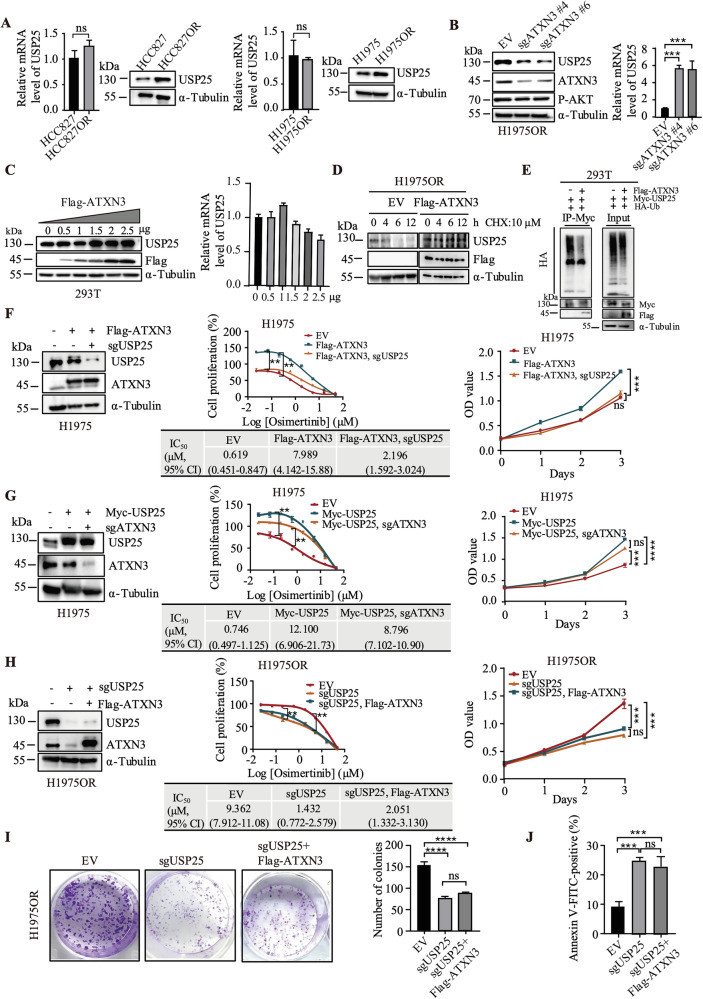
ATXN3 stabilizes USP25 to drive Osimertinib resistance in lung adenocarcinoma. **A** USP25 mRNA and protein levels were measured by Q-PCR and Western Blot in HCC827/H1975 and HCC827OR/ H1975OR cells. **B** H1975OR cells were transfected with the control EV or ATXN3 sgRNAs, cellular extracts were collected for Western Blot with the indicated antibodies and Q-PCR of USP25. **C** Dose-dependent ATXN3 overexpression (OE) in HEK293T cells, cellular extracts were collected for Western Blot with indicated antibodies and Q-PCR of USP25. **D** Cycloheximide chase assay was measured in H1975OR-ATXN3-OE cells, and cellular extracts were collected for Western Blot with indicated antibodies. **E** HEK293T cells co-transfected with HA-ubiquitin (Ub), Myc-USP25 and Flag-ATXN3. Cellular extracts were immunoprecipitated with anti-Myc antibody, followed by WB with the indicated antibodies. **F** H1975 co-transfected with Flag-ATXN3 and sgUSP25. Cellular extracts were collected for Western Blot with the indicated antibodies. The sensitivity to Osimertinib and proliferation ability were measured by CCK8. **G** H1975 co-transfected with sg-ATXN3 and Myc-USP25. Cellular extracts were collected for Western Blot with the indicated antibodies. The sensitivity to Osimertinib and proliferation ability were measured by CCK8. **H** H1975OR co-transfected with Flag-ATXN3 and sgUSP25. Cellular extracts were collected for Western Blot with the indicated antibodies. The sensitivity to Osimertinib and proliferation ability were measured by CCK8. **I** H1975OR transfected with sgUSP25 or/and/or Flag-ATXN3. Colony formation assay and Quantitative analysis were measured. **J** Apoptosis rate was established for indicated cells treated with Osimertinib (1 μM) for 48 h by flow cytometry analysis of Annexin-V-PI dual staining. **B**, **F**, **G**, **H**, **I** *n* = 3; Mean ± SEM; Analysis of variance (ANOVA) with Tukey’s multiple-comparison test. IC_50_ values were estimated using nonlinear regression and are presented with 95% confidence intervals. Differences in IC_50_ values between groups were assessed using an extra sum-of-squares F test. *p < 0.05; **p < 0.01; ***p < 0.001; ****p < 0.0001. EV: Empty vector.

Comparative analysis of Osimertinib-sensitive and -resistant lung adenocarcinoma cells revealed elevated USP25 protein levels in resistant cells without corresponding mRNA upregulation (Fig. 4A). Osimertinib treatment of sensitive cells induced dose- and time-dependent increases in ATXN3 and USP25 protein levels (Fig. S3A–C). While ATXN3 was transcriptionally and translationally upregulated, USP25 accumulation occurred exclusively at the protein level (Fig. S3D), suggesting post-translational regulation. Given ATXN3’s deubiquitinase activity, we hypothesized that the ubiquitin-mediated degradation of USP25 protein was suppressed. Consistently, ATXN3 depletion in H1975OR cells reduced USP25 protein level despite increased mRNA (Fig. 4B), whereas ATXN3 overexpression in 293 T cells elevated USP25 protein without altering mRNA (Fig. 4C). Cycloheximide chase assays confirmed that ATXN3 overexpression extended USP25’s half-life (Fig. 4D). Ubiquitination assays further demonstrated that ATXN3 overexpression suppressed USP25 polyubiquitination (Fig. 4E). However, ATXN3^C14A^ with deubiquitination enzyme active site mutations could not reduce the ubiquitination level of USP25 (Fig. S3E). These results demonstrated that ATXN3 is a critical stabilizer of USP25. To delineate the functional hierarchy, USP25 depletion abolished ATXN3-driven Osimertinib resistance and proliferation in H1975 cells (Fig. 4F). Conversely, ATXN3 depletion cannot abolished USP25-driven Osimertinib resistance and proliferation in H1975 cells (Fig. 4G). Critically, in USP25-depletion H1975OR cells, ATXN3 overexpression failed to restore resistance, proliferation, clonogenicity, or suppression of apoptosis (Fig. 4H–J). Together, these findings identify USP25 as the dominant downstream effector of ATXN3-mediated Osimertinib resistance in lung adenocarcinoma.

### The ATXN3-USP25-TRMT1 axis sustains resistance by attenuating ROS accumulation

**Fig. 5:**
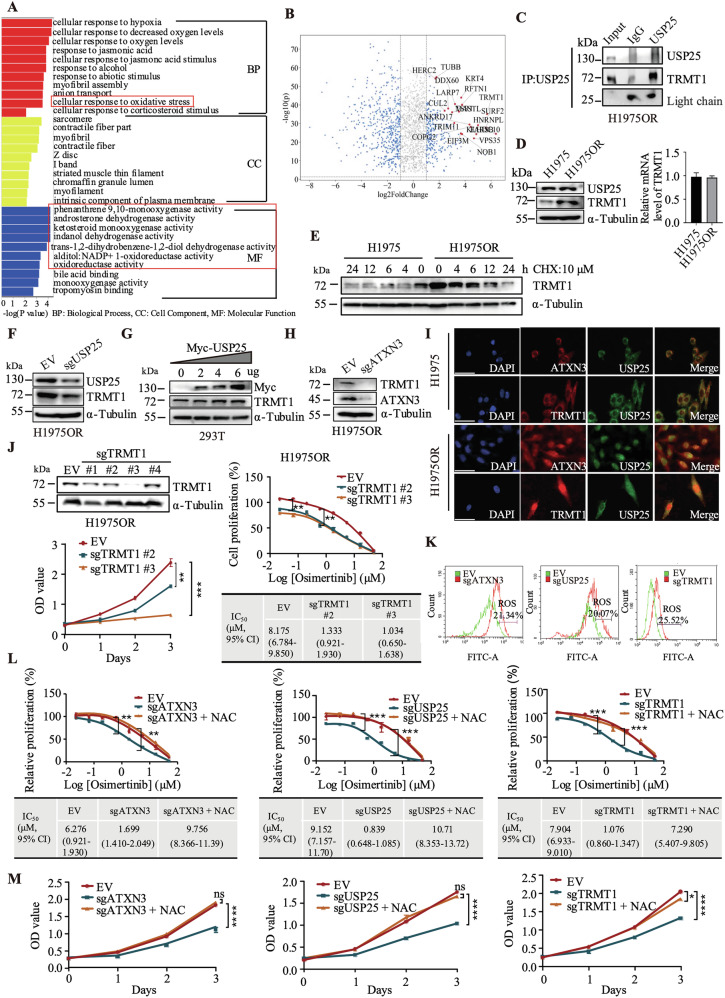
USP25 stabilizes TRMT1 to reduce ROS and drive Osimertinib resistance in lung adenocarcinoma. **A** Gene Ontology (GO) enrichment analysis of differentially expressed genes in USP25-KO H1975OR cells. **B** Volcano plot displaying differentially expressed proteins from proteomic analysis of USP25 interactors in H1975OR cells. **C** Co-IP analysis of the association between USP25 and TRMT1 in H1975OR cells and lysates immunoprecipitated with antibodies against the indicated proteins. **D** USP25 and TRMT1 protein levels were measured by Western Blot, and mRNA levels of TRMT1 were measured by Q-PCR. **E** Cycloheximide chase assay was measured in H1975 and H1975OR cells, and TRMT1 protein levels were measured by Western Blot. **F** H1975OR cells were transfected with the control EV or USP25 sgRNAs, and USP25 and TRMT1 protein levels were measured by Western Blot. **G** Dose-dependent Myc-USP25 were overexpression in HEK293T cells Myc-USP25 and TRMT1 protein levels were measured by Western Blot. **H** H1975OR cells were transfected with the control EV or ATXN3 sgRNAs, TRMT1 and ATXN3 protein levels were measured by Western Blot. **I** Confocal microscopic analysis of ATXN3/TRMT1 with USP25 subcellular localization in H1975 and H1975OR cells. Representative images were shown. Scale bars: 20 µm. **J** H1975OR cells were transfected with the control EV or TRMT1 sgRNAs. TRMT1 protein levels were measured by Western Blot. The sensitivity to Osimertinib and proliferation ability were measured by CCK8. **K** DCFDA staining was used to analyze the level of ROS in cells in H1975OR-ATXN3/USP25/TRMT1-KO cells. **L** Sensitivity to Osimertinib of H1975OR-ATXN3/USP25/TRMT1-KO cells was measured by CCK8 with or without NAC. **M** Proliferative ability of H1975OR-ATXN3/USP25/TRMT1-KO cells was measured by CCK8 with or without NAC. **J**, **L**, **M** *n* = 3; Mean ± SEM; Analysis of variance (ANOVA) with Tukey’s multiple-comparison test. IC_50_ values were estimated using nonlinear regression and are presented with 95% confidence intervals. Differences in IC_50_ values between groups were assessed using an extra sum-of-squares F test. *p < 0.05; **p < 0.01; ***p < 0.001; ****p < 0.0001. EV: Empty vector.

In order to investigate the specific mechanism by which the ATXN3-USP25 axis causes Osimertinib resistance in lung adenocarcinoma, we conducted RNA sequencing analysis in H1975OR and H1975OR-sgUSP25 cells. The results showed significant downregulation of oxidative stress response pathways and redox enzyme activity (Fig. 5A). Additionally, IP-MS identified TRMT1, a tRNA methyltransferase critical for oxidative stress survival, as a USP25 interactor (Fig. 5B) [25]. Co-IP confirmed endogenous USP25-TRMT1 binding in resistant cells (Fig. 5C). TRMT1 protein levels were elevated in Osimertinib-resistant cells despite unchanged mRNA (Fig. 5D, Fig. S4A) and dynamically upregulated following Osimertinib treatment (Fig. S4B), suggesting post-translational stabilization. Cycloheximide chase assays demonstrated a 2.3-fold longer TRMT1 half-life in resistant compared to sensitive cells (Fig. 5E). USP25 depletion reduced TRMT1 protein (Fig. 5F), while USP25 overexpression in HEK293T cells increased TRMT1 levels dose-dependently (Fig. 5G). Ubiquitination experiments demonstrated that overexpression of USP25 reduced the ubiquitination level of TRMT1 and increased the stability of the TRMT1 protein (Fig. S4C, D). Similarly, ATXN3 depletion diminished TRMT1 (Fig. 5H), and immunofluorescence revealed co-localization of ATXN3 or TRMT1 with USP25 (Fig. 5I). Furthermore, we observed that TRMT1 exhibits more nuclear localization signals in 1975OR cells (Fig. 5I). Functionally, TRMT1 depletion in H1975OR cells enhanced Osimertinib sensitivity and suppressed proliferation (Fig. 5J). Depletion of TRMT1, USP25, or ATXN3 increased intracellular ROS by more than 20% (Fig. 5K). Notably, the ROS scavenger NAC rescued Osimertinib resistance and proliferation in TRMT1/USP25/ATXN3-depletion cells (Fig. 5L, M). These results establish the ATXN3-USP25-TRMT1 axis as a critical ROS-buffering mechanism that sustains Osimertinib resistance in lung adenocarcinoma.

### TRMT1 deficiency disrupts tRNA m²_2_G modification and redox enzyme output

**Fig. 6:**
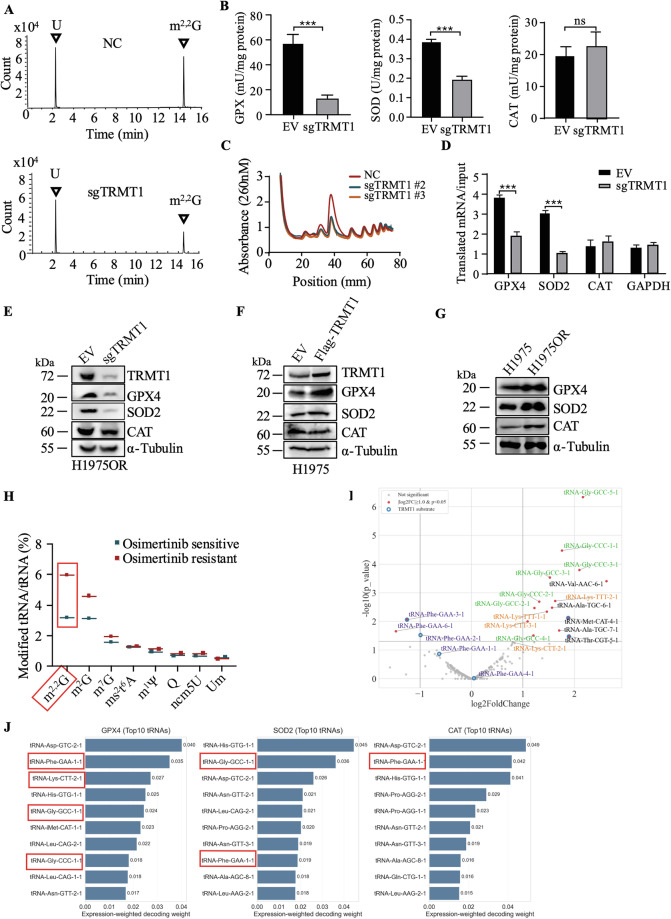
TRMT1-mediated tRNA m²_2_G modifications enhance redox enzyme translation to suppress ROS. H1975OR cells were transfected with the control EV or TRMT1 sgRNA. The m²_2_G modification levels were measured by tRNA mass spectrometry (**A**). The activity of GPX, SOD and CAT were assessed using the Activity Kit (**B**). The global translation level of the affected proteins was evaluated by Polysome profiling (**C**). mRNA level of indicated genes was detected by Q-PCR and the protein level was detected by Western Blot (**D**, **E**). **F** H1975 cells were transfected with the control EV or Flag-TRMT1, and the protein level of the indicated genes was detected by Western Blot. **G** Western Blot analyzed GPX4, SOD2, and CAT expression levels in H1975 and H1975OR cells. **H** tRNA MS detected differences in tRNA modification levels between sensitive and resistant cells. **I** H1975 OR cells were transfected with the control EV or sg-TRMT1. The volcano plot of differentially expressed tRNAs was analyzed by tRNA-seq. **J** Bar plots showing the top 10 tRNAs contributing to the expression-weighted codon decoding capacity for GPX4, SOD2, and CAT. The decoding weight was calculated by integrating tRNA expression levels with codon usage frequencies of each gene, reflecting the relative contribution of individual tRNAs to the translation of these antioxidant enzymes. (**B**, **D**) *n* = 3; Mean ± SEM; Analysis of variance (ANOVA) with Tukey’s multiple-comparison test. *p < 0.05; **p < 0.01; ***p < 0.001; ****p < 0.0001. EV: Empty vector.

TRMT1 is currently the only known enzyme responsible for m²_2_G formation at position 26 in human tRNAs [26].TRMT1 depletion in Osimertinib-resistant cells reduced m²_2_G levels by 63% (Fig. 6A). Superoxide dismutase (SOD), glutathione peroxidase (GPX), and catalase (CAT) are three important antioxidant enzymes that play a role in combating oxidative stress within cells. Concomitantly, TRMT1 depletion diminished GPX and SOD activity (*P* < 0.001), while CAT activity remained unchanged (Fig. 6B). Polysome profiling revealed a decrease in global translation efficiency in TRMT1-depletion cells (Fig. 6C), with pronounced deficits in *GPX4* and *SOD2* mRNA ribosome occupancy (Fig. 6D). Western Blot confirmed selective depletion of GPX4 and SOD2 protein levels (Fig. 6E), while TRMT1 overexpression restored both (Fig. 6F). In addition, GPX4, SOD2, and CAT are highly expressed in H1975OR cells and HCC827OR cells (Fig. 6G and S4E). The CAT protein was upregulated in drug-resistant cells (Fig. 6G and S4E), but not in TRMT1 overexpressing cells (Fig. 6F), indicating that TRMT1 is not the main factor regulating CAT. These findings established that TRMT1-mediated m²_2_G modification selectively regulates the expression levels of redox enzymes. Consistently, comparative analysis of Osimertinib-sensitive and -resistant cells observed a 1.8-fold increase in tRNA m²_2_G modifications (Fig. 6H and S5).

In order to clarify how TRMT1 enhances the translation efficiency of specific redox enzymes by modulating the tRNA m²_2_G modification, tRNA-seq was conducted in TRMT1-depletion H1975OR cells, and the results revealed selective depletion of 15 tRNA isoforms (5.7% of 265 total tRNA isodecoders detected in the tRNA-seq). The significantly downregulated tRNAs are several isodecoders of tRNA-Phe. The significantly up-regulated tRNAs include several isodecoders of tRNA-Gly and tRNA-Lys. (Fig. 6I).

We conducted codon usage analysis using the CDS of MANE Select (major transcripts) and calculated the decoding contribution ratio of each tRNA within the context of tRNA expression levels obtained from tRNA-seq to reflect the decoding dependency of the proteins on specific tRNAs. When considering only TRMT1 substrate tRNAs, there was no significant difference in the overall dependency of GPX4, SOD2, and CAT proteins on TRMT1 substrate tRNAs (Tables S2–3). However, when we analyzed the top 10 tRNAs upon which GPX4, SOD2, and CAT decoding depend, we found that the tRNAs with strong decoding dependency for GPX4—namely tRNA-Phe, tRNA-Lys, and tRNA-Gly—are all tRNAs that change significantly after TRMT1 knockout. SOD2 also exhibited strong decoding dependency on tRNA-Phe and tRNA-Gly. In contrast, among the tRNAs with strong decoding dependency for CAT, only tRNA-Phe is affected by TRMT1 (Fig. 6J). These results explain why GPX4 and SOD2 are significantly regulated by TRMT1, while CAT is less affected. The balance between the tRNA pool and codon usage in the transcriptome is crucial for maintaining high expression of specific genes [27]. This also suggests that we should not focus solely on TRMT1 substrate tRNAs; some tRNAs that are not TRMT1 substrates but change significantly after TRMT1 knockout also influence protein decoding. This codon bias aligns with TRMT1’s preferential impact on GPX4/SOD2 translation.

### Pharmacological targeting of the ATXN3-USP25-TRMT1 axis reversed Osimertinib resistance in preclinical models

**Fig. 7:**
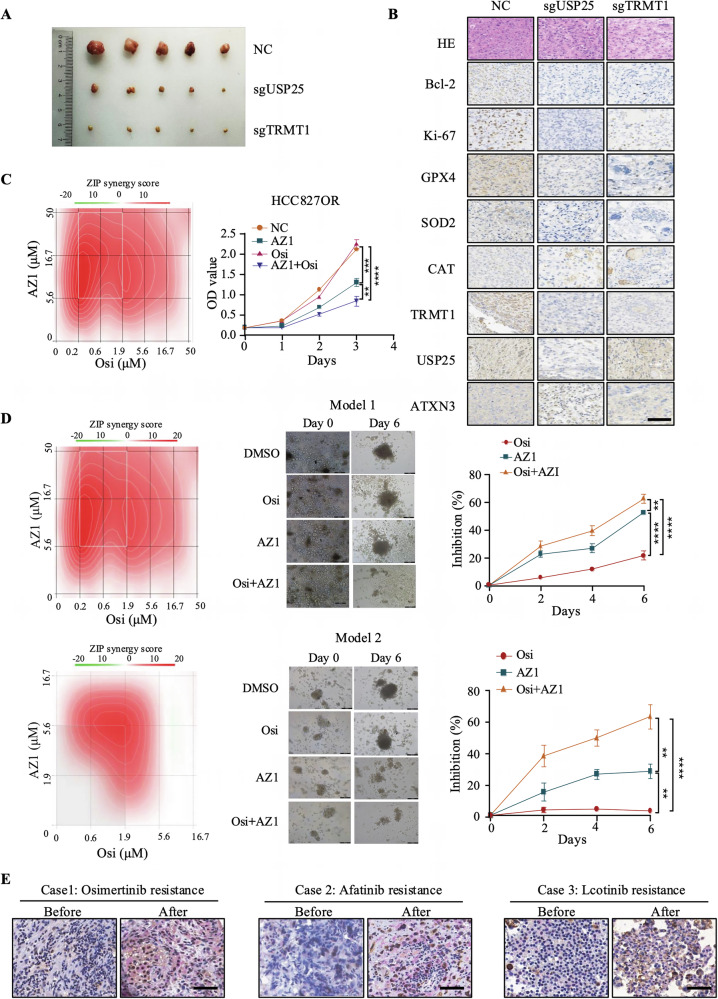
Therapeutic targeting of the ATXN3-USP25-TRMT1 axis overcomes Osimertinib resistance in vitro, in vivo, and in patient-derived models. **A** Mice were subcutaneously injected with HCC827OR parental (NC), USP25-KO (sgUSP25), and TRMT1-KO (sgTRMT1) cells, and tumors were removed and recorded. *n* = 5. **B** Representative images of tumor tissues stained with HE and IHC for indicated proteins. HCC827OR cells (**C**) and Organoid Model (**D**) were cultured in the control medium or in the presence of Osi and/or AZ1 for 72 h. The cell viability was determined by the CCK-8 kit. Data were analyzed online (https://synergyfinder.fimm.fi). The growth curves were displayed of HCC827OR cells treated with DMSO, AZ1 (16 μM), Osi (0.6 μM), and the combination of AZ1 (16 μM) + Osi (0.6 μM) (**C**). Representative images were shown, and growth inhibition rates were measured of organoid Models treated with DMSO, Osi (1.9 μM), AZ1 (5.6 μM), and Osi (1.9 μM) + AZ1 (5.6 μM). Scale bar: 200 μm (**D**). **E** Representative IHC images of USP25 and TRMT1 expression levels before and after resistance to Osimertinib, Afatinib, and Lcotinib (brown for TRMT1, pink for USP25, and purple-blue for hematoxylin staining; Scale bar: 50 μm). **C**, **D** *n* = 3; Mean ± SEM; Analysis of variance (ANOVA) with Tukey’s multiple-comparison test *p < 0.05; **p < 0.01; ***p < 0.001; ****p < 0.0001.

To determine whether targeting the ATXN3-USP25-TRMT1 axis has anti-tumor activity, we conducted a Tumor Xenograft experiment in BALB/c nude mice. The results showed that compared to the control group, USP25 or TRMT1 depletion in H1975OR reduced tumor volume by 78% and 82%, respectively (Fig. 7A), with immunohistochemistry confirming decreased Ki-67 and Bcl-2 in USP25 or TRMT1 depletion tumors (Figs. 7B and S6A). GPX4 and SOD2 protein depletion in tumors mirrored cellular observations (Fig. 7B and S6A), while CAT remained unaffected. USP25 or TRMT1 depletion destabilized TRMT1 (USP25-KO) without altering ATXN3 levels in vivo (Fig. 7B and S6A), reinforcing the axis hierarchy. Additionally, we observed the USP25 inhibitor AZ1 suppressed the proliferation of Osimertinib-resistant cells by 58% as a monotherapy and synergized with Osimertinib (Fig. 7C). Similarly, in organoids derived from three Osimertinib-resistant patients. AZ1 alone reduced viability by 21–42% and achieved >50% cell death in combination with Osimertinib (Fig. 7D and S6B). In order to investigate whether the resistance mechanisms of tumors to Osimertinib are also applicable to other EGFR-TKIs, we chose two other drugs (Afatinib and Loctinib), resistant patient samples for validation. Similarly, we observed the increase in USP25 and TRMT1 protein after resistance emergence in paired pre-/post-resistance biopsies from NSCLC patients (Fig. 7E), implicating this axis in broad adaptive responses to EGFR tyrosine kinase inhibitors. Finally, we evaluated the association between baseline ATXN3, USP25, and TRMT1 expression levels and progression-free survival (PFS) in patients treated with Osimertinib. The results showed that patients with lower overall ATXN3/USP25/TRMT1 expression had a longer PFS compared with those with high expression (Fig. S7). Compared to evaluating ATXN3, USP25, and TRMT1 individually, assessing the overall levels of the three can reduce individual differences and more objectively predict the patient’s response to Osimertinib treatment (Figs. 1F and S7). Altogether, these results suggest that targeting ATXN3-USP25-TRMT1 axis is expected to reverse Osimertinib resistance in clinical.

## Discussion

The emergence of resistance to Osimertinib represents a critical barrier to durable responses in EGFR-mutant lung adenocarcinoma. This study uncovers a previously unrecognized resistance mechanism governed by an ATXN3-USP25-TRMT1 signaling axis that couples deubiquitination-driven protein stabilization to tRNA epitranscriptomic reprogramming, enabling cancer cells to evade Osimertinib-induced oxidative stress. Our findings position tRNA modification as a novel driver of therapy resistance and nominate the ATXN3/USP25/TRMT1 axis as a therapeutically actionable target.

**Fig. 8:**
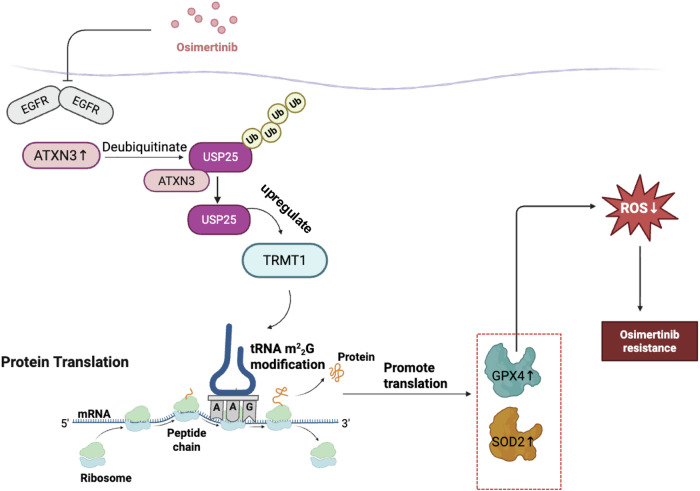
Proposed model of TRMT1-Mediated tRNA m²_2_G Modification Drives Osimertinib Resistance via the ATXN3/USP25 Axis in Lung Adenocarcinoma.

Central to this mechanism is the dynamic upregulation of ATXN3 during Osimertinib treatment. ATXN3, a DUB with context-dependent roles in cancer [28–31], stabilizes USP25 through direct deubiquitination, forming a positive feedback loop that amplifies DUB activity. USP25, in turn, enhances TRMT1 protein levels, promoting m²_2_G tRNA modifications. This epitranscriptomic rewiring selectively enriches the translation of GPX4, SOD2, and other redox-regulating enzymes, effectively scavenging lethal ROS generated by Osimertinib (Fig. 8). Our data align with emerging evidence that tRNA modifications fine-tune the translational efficiency of stress-response genes in cancer [32], but to our knowledge, this is the first report linking tRNA epitranscriptomics to EGFR-TKI resistance. The translational selectivity for redox enzymes, rather than global protein synthesis, suggests a precise adaptation strategy that balances survival with metabolic cost, a hallmark of persistent minimal residual disease.

Our work diverges from prior models of Osimertinib resistance, which largely focus on genomic alterations (e.g., EGFR C797S, MET amplification) [33]. Instead, we reveal that non-genetic, post-translational regulation of tRNA machinery can sustain therapeutic evasion. Notably, this pathway operates across multiple resistance contexts: ATXN3 amplification occurs in ~8% of Osimertinib-resistant LUAD patients (cBioPortal), while USP25 overexpression correlates with poor survival in TCGA-LUAD, suggesting broader relevance. The clinical tractability of this axis is underscored by our demonstration that AZ1, a brain-penetrant USP25 inhibitor, restores Osimertinib sensitivity in PDX models and patient-derived organoids without overt toxicity. This positions USP25 inhibition as a promising adjunct to Osimertinib, particularly given the paucity of targets in resistance settings lacking actionable mutations.

Key questions remain. First, how does TRMT1-mediated m²_2_G modification preferentially enhance translation of redox enzymes? Ribosome profiling in TRMT1-deficient cells could reveal codon-specific translational biases linked to m²_2_G modified tRNAs. Second, while ATXN3/USP25 drives resistance in our models, their roles in normal tissue homeostasis demand careful evaluation to anticipate on-target toxicities. Third, we did not directly detect the effects of ATXN3/USP25 on tRNA m^2^_2_G modification. Finally, the interplay between tRNA epitranscriptomic changes and other resistance pathways warrants exploration.

In conclusion, we propose a paradigm in which DUB-regulated tRNA modification serves as a molecular rheostat for redox adaptation under therapeutic pressure. Pharmacologically disrupting this axis with USP25 inhibitors represents a translatable strategy to overcome Osimertinib resistance. Our findings further nominate tRNA epitranscriptomics as a frontier for understanding cancer evolution and a new therapeutic dimension in the post-genomic era.

## Supplementary information

Supplemental Figures and Tables

Silver-stained gel, full western blots and quantification

## Data Availability

Data is provided within the manuscript or supplementary information files. MS raw data upload to iProx: PXD078535.
