# Supplementary material for: TRMT1-mediated tRNA m22G modification drives Osimertinib resistance via the ATXN3/USP25 axis in lung adenocarcinoma
Source: Cell Death Dis. 2026 Jun 29;17(1):660. doi: 10.1038/s41419-026-09039-8 (PMC13408171; doi:10.1038/s41419-026-09039-8)
Supplement: Supplementary file 1 — Supplemental Figures and Tables [file 41419_2026_9039_MOESM1_ESM.pdf]

Supplemental Figures

TRMT1-Mediated tRNA $m^2{}_2G$ Modification Drives Osimertinib Resistance via the ATXN3/USP25 Axis in Lung Adenocarcinoma

Supplementary Figure S1

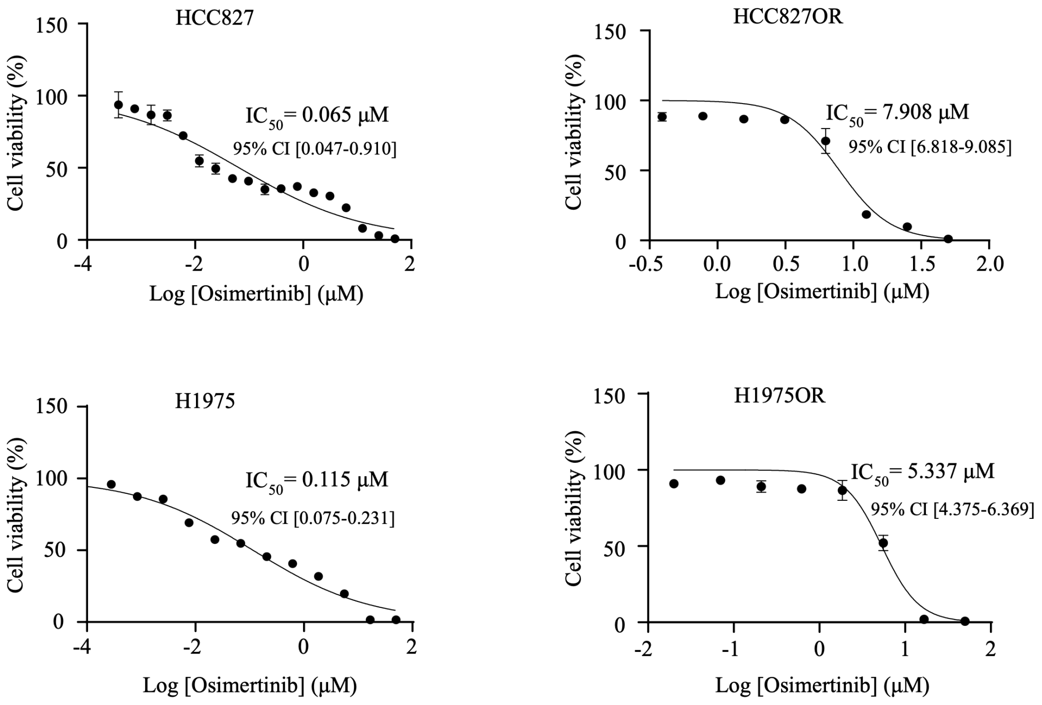

Figure S1, Related to Figure 1. CCK-8 assays measuring the IC_{50} values and 95%CI of Osimertinib-sensitive (HCC827, H1975) and Osimertinib-resistant (HCC827OR, H1975OR) cell lines. n=3; IC_{50} values were estimated using nonlinear regression and are presented with 95% confidence intervals.

Supplementary Figure S2

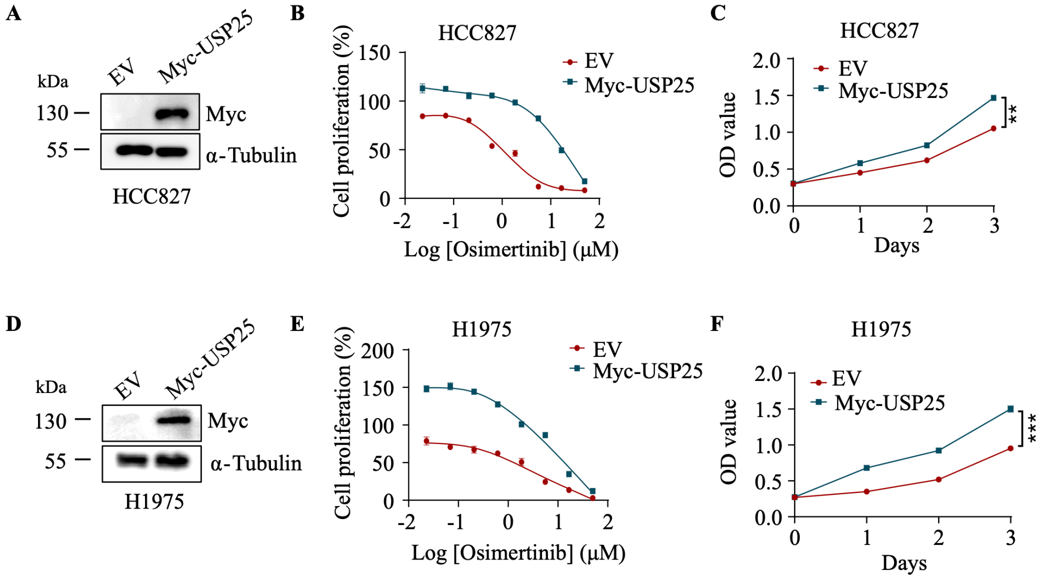

Figure S2. Related to Figure 3. A-F, HCC827 and H1975 cells were transfected with the control EV or Myc-USP25. Protein levels of USP25 were detected by Western Blot **(A, D)**; The sensitivity to Osimertinib and proliferation ability were detected by CCK8 **(B-C, E-F)**. n=3; Mean ± SEM; Student's t test. *p<0.05; **p<0.01; ***p<0.001; ****p<0.0001. EV: Empty vector.

Supplementary Figure S3

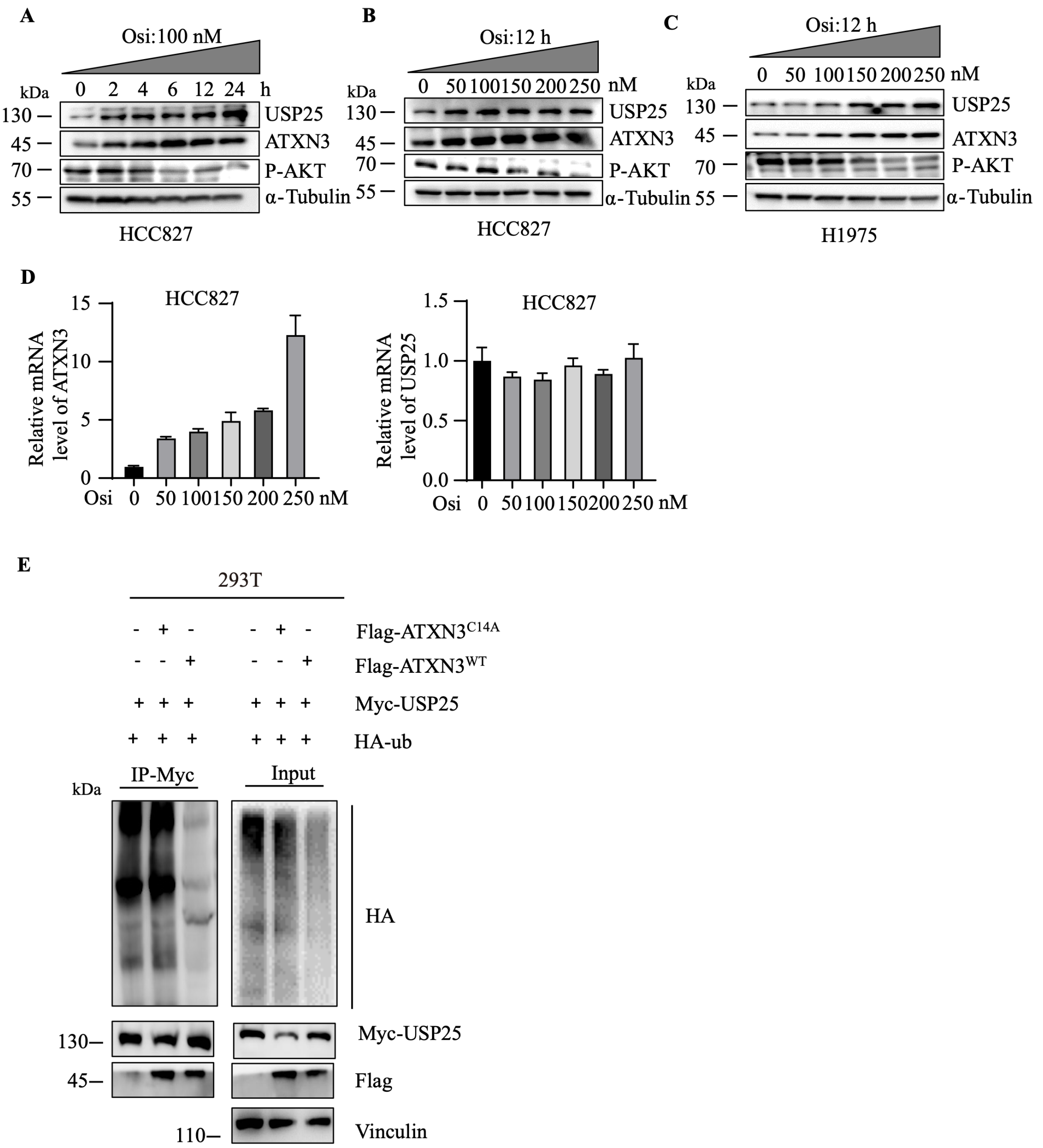

Figure S3. Related to Figure 4. A, Treatment of HCC827 cells with 100 nM Osimertinib for 0, 2, 4, 6, 12, and 24 h, cellular extracts were collected for Western Blot with indicated antibodies. **B-D,** Treatment of HCC827 and H1975 cells with different concentrations of Osimertinib for 12 h. Cellular extracts were collected for Western Blot with indicated antibodies **(B-C)** and Q-PCR **(D)**. **E,** HEK293T cells co-transfected with HA-ubiquitin (Ub), Myc-

USP25 and Flag-ATXN3^{WT} or Flag-ATXN3^{C14A}. Cellular extracts were immunoprecipitated with anti-Myc antibody followed by WB with indicated antibodies.

Supplementary Figure S4

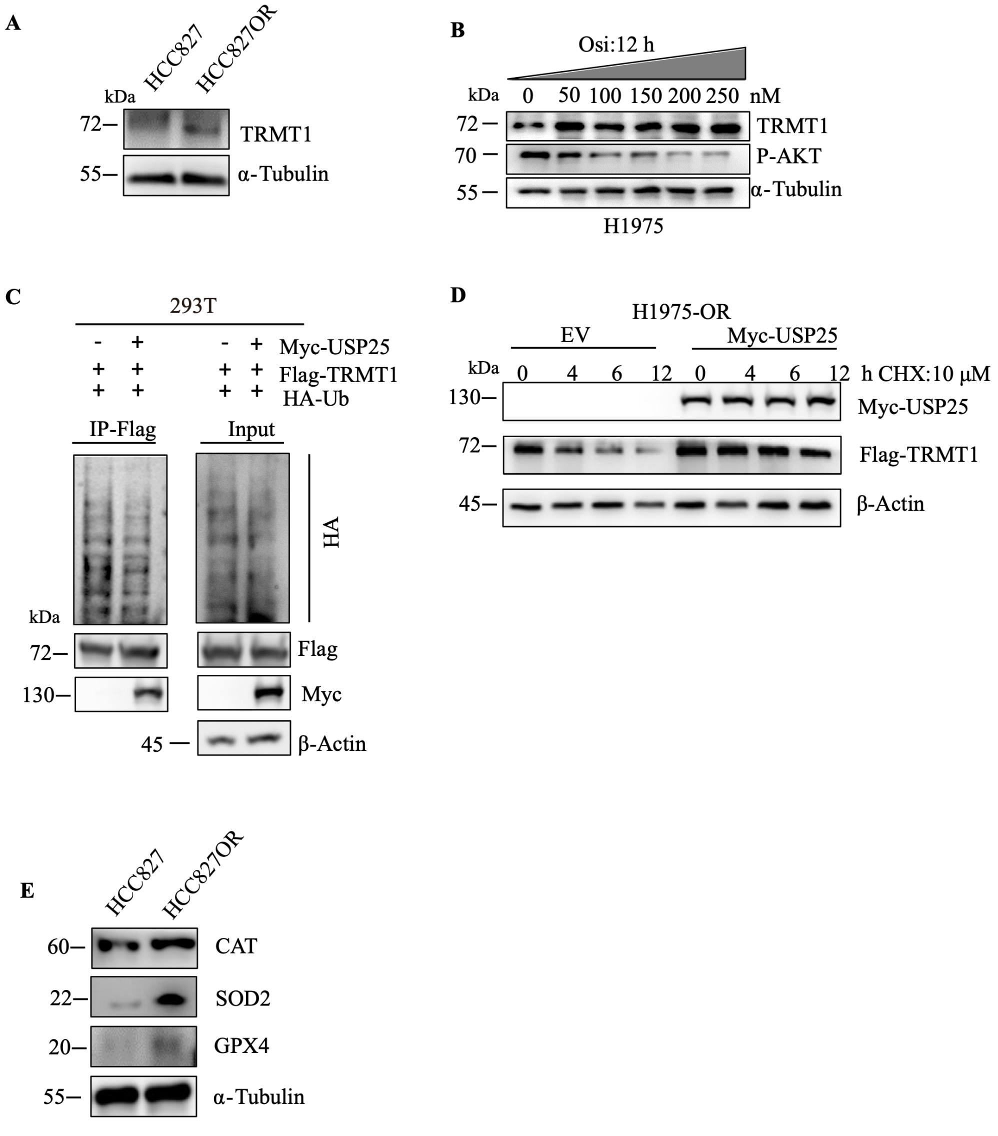

Figure S4. Related to Figure 5- Figure 6. A, Western Blot detect TRMT1 in HCC827 and HCC827OR cells. **B,** Treatment of H1975 cells with different concentrations of Osimertinib for 12 h, cellular extracts were collected for Western Blot with the indicated antibodies. **C,** HEK293T cells co-transfected

with HA-ubiquitin (Ub), Myc-USP25 and Flag-TRMT1. Cellular extracts were immunoprecipitated with anti-Myc antibody followed by WB with indicated antibodies. **D,** Cycloheximide chase assay were measured in H1975OR-USP25-OE cells, and cellular extracts were collected for Western Blot with the indicated antibodies. **E,** Western Blot detects CAT, SOD2, GPX4 in HCC827 and HCC827OR cells.

Supplementary Figure S5

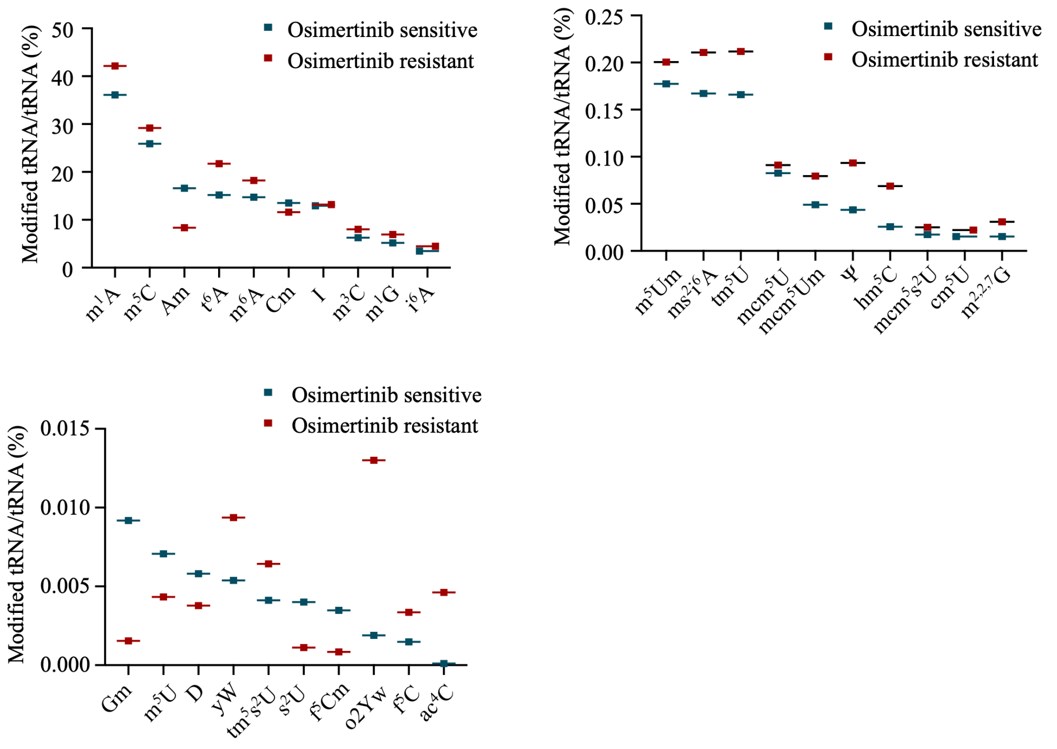

Figure S5. Related to Figure 6. tRNA mass spectrometry analysis of tRNA modification levels in Osimertinib-sensitive and resistant cells.

Supplementary Figure S6

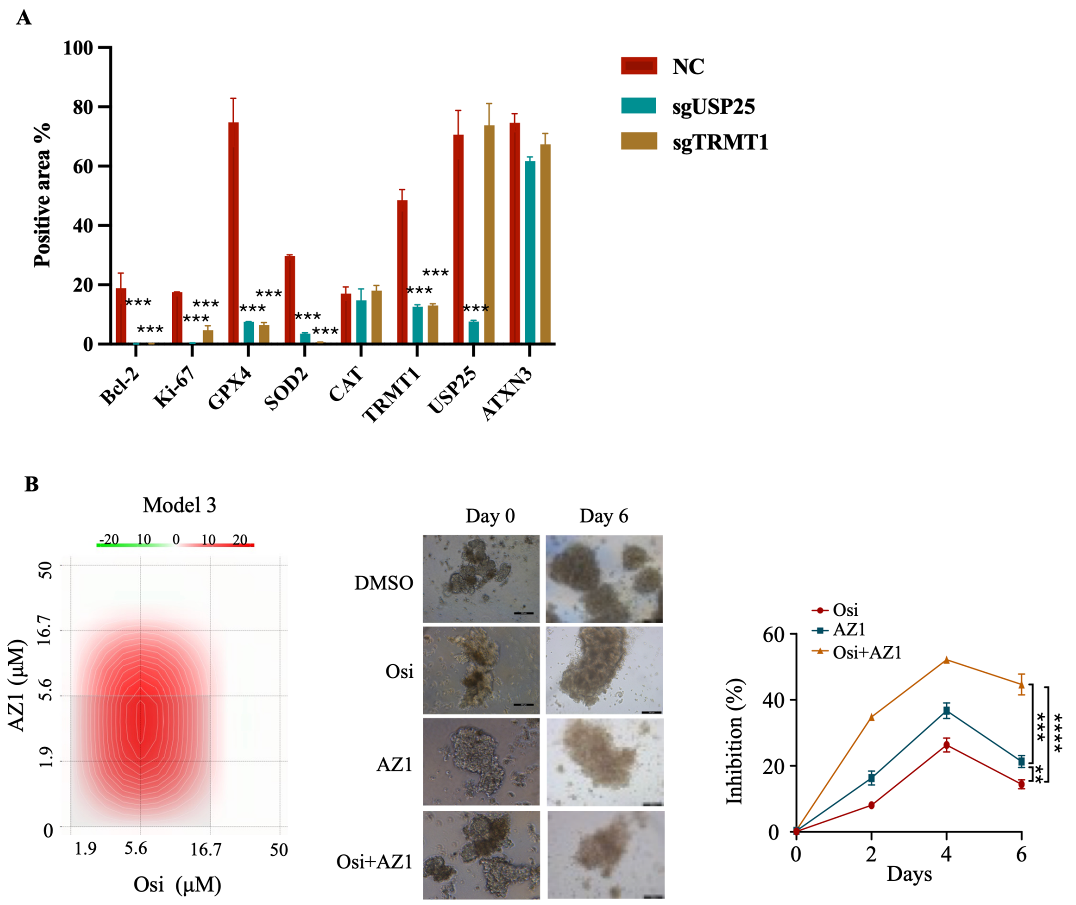

Figure S6. Related to Figure 7. A, Quantitative analysis of immunohistochemistry images (Figure 7B). **B,** Organoid Model derived from patients were cultured in the control medium or in the presence of Osi and/or AZ1 for 72 h. Representative images were shown and growth inhibition rate were measured of organoid Model treated with DMSO, Osi (5.6 μM), AZ1 (5.6 μM), and Osi (5.6 μM) + AZ1 (5.6 μM). Scale bar: 200 μm. n=3; Mean ± SEM; Analysis of variance (ANOVA) with Tukey's multiple-comparison test. *p<0.05; **p<0.01; ***p<0.001; ****p<0.0001.

Supplementary Figure S7

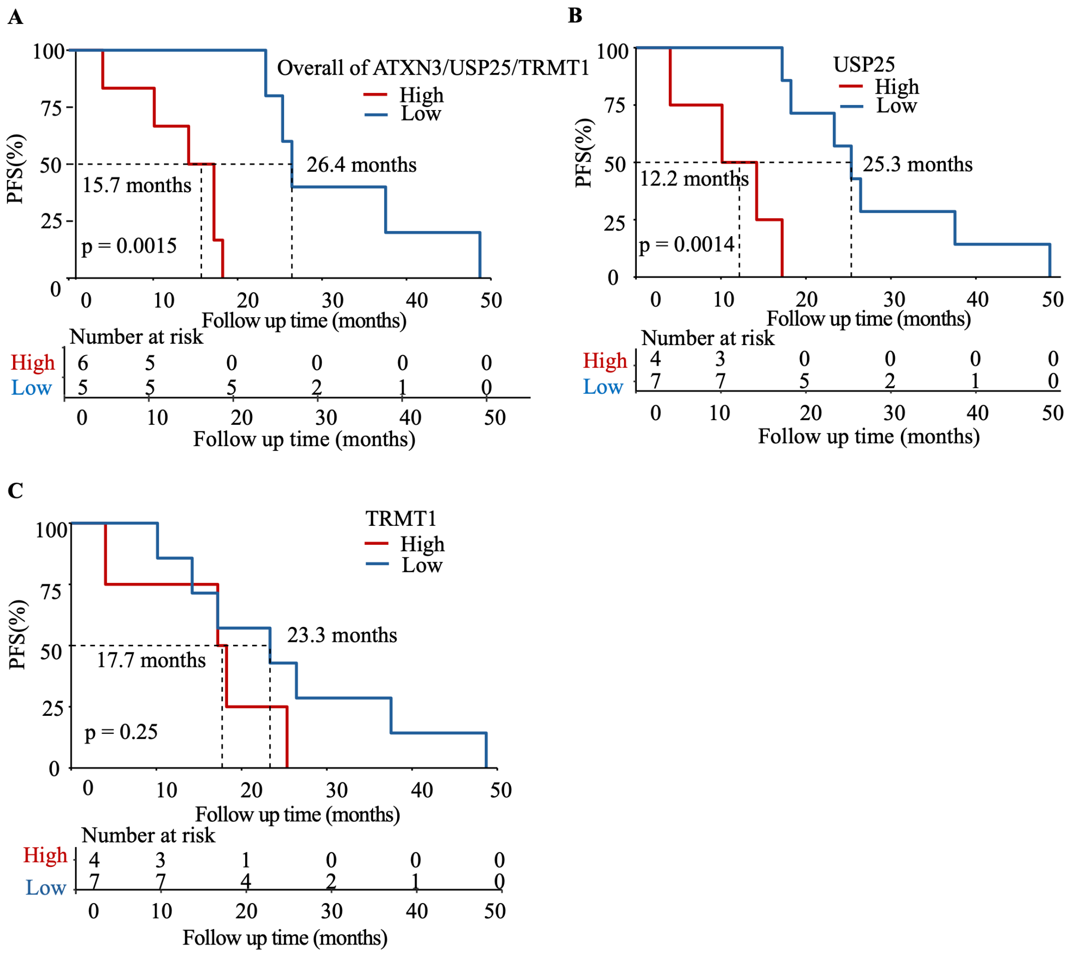

Figure S7. Related to Figure 7. A, Kaplan–Meier Plot of Progression-Free Survival treated with Osimertinib based on the overall expression levels of baseline ATXN3/USP25/TRMT1. **B,** Kaplan–Meier Plot of Progression-Free Survival treated with Osimertinib based on the expression levels of baseline USP25. **C,** Kaplan–Meier Plot of Progression-Free Survival treated with Osimertinib based on the expression levels of baseline TRMT1.

Supplementary Tables

Table S1: Primers required for the experiment.

Name	Forward （5'-3'）	Reverse （5'-3'）
USP1	AACTGCCATCATTATACTG	TTGTGCTCCATTCTTCTA
USP2	TGCTGAGACCCGACATCACT	TGGGGTCTATCCGGTAGCTA
USP3	CTGTTATCGCTGTGATGAT	CCAAGTTCTGTAAGTGTTCT
USP4	GTCTTGTGAACTCCTATG	CAGATTCTTGCTCATCAT
USP5	TGGCTATTGGTGTTGAAG	GGCAATCTCCAGGTAATC
USP6	ATGTGGAACTGAGAAGAA	GCAATGATGTAACCTGTAA
USP7	GCAAGTGCAGATAGTCGCAGAGGAC	CCATGGTCTGAGAGAGGCTCTGAAC
USP8	ACTATACACAATGATGACGGATA	TGGACTGATGGCTTCTTC
USP9X	AGGTGGTGGAATGCTTAT	GAGGTCTGGTGGTGATAG
USP9Y	CTCAGTATCAACAGAATAATC	TCTTCATTGCCTTCATAA
USP10	GAATCTGTCCAAGGTTATAC	CCACCAGTCTTCTCATAA
USP11	AGTAGAACTGCTGCTTGT	ATAGAATCGGTATGGCTGAA
USP12	TGCGTATAAGAGTCAACCT	GAGTGGCTATGCTATGGA
USP13	ACGAGCAACGAATAATAAC	TTCTCCATCTCAATCACAA
USP14	AAATGGCTTCAGCGCAGTAT	TTCACCTTTCTCGGCAAACT
USP15	GACAGGTATTAGTGATAGA	GATAGAGATGAAGGAGAG
USP16	AGAACCTGAGTTAAGTGATG	CAATCTTGCTGTCCTCTC
USP17L	AACAAGATTGCCAAGAATG	TAGAGGACATAGACGAGAG
USP18	AATGGTTCTGCTTCAATG	CCAGTGGTAGTTAGGATT
USP19	TGGACCTGAGCAAGTTCT	ATAGTGGTTGATGACAGCATATAG
USP20	TATGTTGGCTGCGGAGAA	AGGTTCACGGTCAAGTTGT
USP21	AGAACCTGAGTTAAGTGATG	CAATCTTGCTGTCCTCTC
USP22	GAGAGCAGGATGAATGGA	AACAGCAAACAGGGAATAC
USP24	TGAGATGCCAGTTATTAGA	AGTTATCCAGCCAAGTAA
USP25	TCTCCTGTTGACGATATTG	TTCTGTTGTGCTTGGTAA
USP27X	ATGTGTAAGGACTATGTATATGAC	GAGGTGGAGGCTTGTAAT
USP28	AATGCTGGACACTATTGG	GGAAGATTCAGTAACAGAGAT
USP29	ATGCTGTTCTCAAGGTAG	AACTCTTCTGCTTACACAT

USP30	ACTGATGATGAGGTCTTAG	TTCCAATGACGAGGTAAT
USP32	ATGTGGAACTGAGAAGAA	GCAATGATGTAACCTGTAA
USP34	GTTGTTGACTATGCTAAT	ACTGAGAAGGATTGTATT
USP35	ATTAGCAGGATGATTGAC	TGAACTTCTTAACAGCAG
USP37	CATCAGTGTTGTCAGTCA	CTCCAGGTCATTGTAAGTAA
USP38	CAGCATATTCCTCTTCAG	ATAGCCAGTCAATCATTC
USP39	GGCATCACTGAGAAGGAA	AAGATTAGATATGGAGGCAACT
USP40	CTCTTCTCAGTTATTATACAC	CAATCTCTTCTTCACTCT
USP41	AATGGTTCTGCTTCAATG	CCAGTGGTAGTTAGGATT
USP42	ATCAGTATTCACAACAAG	ATTGGTAATGGTAGAAGA
USP43	TGGTACAGTTATGATGAC	TGATAGAACAGGATATAAGC
USP44	ACCAGTCACAGATACAGTAG	TTGATGCTCCACCACTTA
USP45	CTGCTCTGCTACTTGTAA	GCCACTATGTTCCACTAT
USP46	CGATGCTTGAACTGTGAA	TGTGTTGCTGAAGTCTCT
USP47	GCTAATGGACTTGACTCT	CACTCTCATCATATTCACTATC
USP48	CACTCTACTTATGTCCAA	ATCAATGTATCGCCTATT
USP49	GCTTGTGACCAGTGTAAC	GAGGTAGTCTGTAGATCATTAAC
USP50	GGATTACCACTGAGACAT	AGACTTCGTTCTTGTAGG
USP51	AAGACAAGCAATCAACCT	CCTGGACAATACAATTCATAA
USP52	AGGTGGTGGATTACTTGAC	GTTAGGTGCTTGGAGGAA
USP53	GCTCCTCAACTAACGATT	CTCATTTGGACAGGTAGAA
USP54	ATTCTTCTCTTGCCTTAA	CTTCTCTTGGTATATGTCT
CYLD	AGGCTTGGAGATAATGATTGG	GCAGAATAAGGTTGAGTCTAAGTA
USPL1	CTCCACATAAGCCTCAGAA	TCCACCGTCAATAGCAATA
UCHL1	CAAGAAGTTAGTCCTAAAGTGTA	GCGTGAATAAGTCCGATT
UCHL3	GGCAATTCGTTGATGTAT	TCTTCCTCTTCTGTTCTG
UCHL5	TTAATAATGCTTGTGCTACTC	CTGATAATGTCTCGCCTAA
BAP1	TATCTTCCTGTTCAAATG	CAATATCATCATCAATCAC
ATXN3	AACATTGCCTGAATAACT	TAGTAACTCCTCCTTCTG
ATXN3L	ACCAATAGAGAAGATGAACA	GAAGCAGGAGTTACACAT
OTUB1	AGGAGTATGCTGAAGATGAC	CTTGCGGATGTACGAGTA
OTUB2	GAGGAGCACAAGTTCAGAA	ACACTGAGCCATCCTTCT

OTUD1	AGATGCTGAATGTGAATA	TAATGAATCATGGTAGACA
OTUD3	TCTGAAGACGACCTGAGAG	CGAAGCACGGCAATTATTG
OTUD4	ATCCAAGCAGTTCTATAATCA	ACTCCTCACTTCTCACAT
HIN1L	GAAGCGATTATAGGAGGAT	AGTTACTTGTGAAGGAGAA
OTUD5	GGACTATCTGATGAAGAATGC	TCCGCTTCCTGTTAATGTA
OTUD6A	AGTAGCATTGAATCTGTC	ACTCCATTCTTTCTCTCT
OTUD6B	CAAGAATGACAAGAAGAG	TAACAGCAACAGAATCTA
YOD1	ATACACAGACAGTAAGAA	TATCATCATTAGAGGAGAA
A20	AATGAGATGAAGGAGAAG	ATTGATGAGATGAGTTGT
OTUD7A	AGCAATTCTAACAGCAATAAC	GTCTTGTCCTTCTCCTTG
OTUD7B	AGCAGACACAGCAGAATA	TCAGTTCATTCCACTCCTT
TRABID	GACTGATTGGCTCTTCCT	TGATGACTTGTATGCTTCTATG
VCPIP1	TGGAGTAGTAACAATGAGA	TGAAGCCTGAATAGAAGA
BRCC3	GATTACTATGGTCACTTG	CATCATCCTCATCAATAG
COPS5	ACTCAGATGCTCAATCAG	TGCGGATATTGTTCTTGT
COPS6	CCCTCTTTCTGAAGTTGA	GCCTCTCCATTGATTATATC
PSMD14	CAATGCTAATATGATGGTCTTA	GTAATGGAGTAATAATGTCTGTT
PSMD7	AAGAATAGTTGGCTGGTA	CGGAATTAGGACAGTATCT
AMSH	GAGTTGAGATTATCCGAATG	AGAGCGTGATATACTTGTT
AMSH-LP	ATGGAGAATGTAGAGGAAT	TTGATAGGAACAGTGAGT
MPND	GCAGCCATCAACAAGTTC	TCAGGTGACTGTGGAAGT
MYSM1	ATTGTATTGGACGGATTC	GTTGGTATGCTTCTACTG
PRPF8	GGATGAAGACTGGAATGAAT	TGGTGTGGAAGATTGTTG
EIF3F	TTCCTGATGAGCCTGGTTA	GGTCATTGATGTTGCTGTTG
EIF3H	GATGGACAGAGTGGATGAA	GACGCTGCTGATACTGAT
JOSD1	GTGGATTGGAGGCGAGAG	AGCAGGAGTTCACAGTTCTT
JOSD2	TGAGATCTGCAAGAGGTT	ATCACATTGACATCATAGTTG
JOSD3	TTGACAGTCGTAGATACA	TTCAGTAATATCCTCTTCTTC

Table S2. The decoding weight of the TRMT1 substrate tRNA.

Gene	Transcript ID	CDS codons (n)	Total TRMT1 substrate tRNA decoding weight
CAT	ENST00000241052.5	527	0.288
GPX4	ENST00000354171.13	197	0.255
SOD2	ENST00000538183.7	222	0.221

Table S3. TRMT1 Predicated Substrate in Human tRNAs. (Based on the GtRNAdb database.)

Isotype	Isodecoder	Predicted modification at position 26	Prediction basis
Ala	tRNA-Ala-AGC-10-1	m2,2G (TRMT1 candidate; G)	Sequence index (pos26)
Ala	tRNA-Ala-AGC-11-1	m2,2G (TRMT1 candidate; G)	Sequence index (pos26)
Ala	tRNA-Ala-AGC-12-1	m2,2G (TRMT1 candidate; G)	Sequence index (pos26)
Ala	tRNA-Ala-AGC-13-1	m2,2G (TRMT1 candidate; G)	Sequence index (pos26)
Ala	tRNA-Ala-AGC-14-1	m2,2G (TRMT1 candidate; G)	Sequence index (pos26)
Ala	tRNA-Ala-AGC-15-1	m2,2G (TRMT1 candidate; G)	Sequence index (pos26)
Ala	tRNA-Ala-AGC-16-1	m2,2G (TRMT1 candidate; G)	Sequence index (pos26)
Ala	tRNA-Ala-AGC-8-1	m2,2G (TRMT1 candidate; G)	Sequence index (pos26)
Ala	tRNA-Ala-AGC-9-1	m2,2G (TRMT1 candidate; G)	Sequence index (pos26)
Arg	tRNA-Arg-ACG-1-1	m2,2G (TRMT1 candidate; G)	Sequence index (pos26)
Arg	tRNA-Arg-ACG-2-1	m2,2G (TRMT1 candidate; G)	Sequence index (pos26)
Arg	tRNA-Arg-CCG-1-1	m2,2G (TRMT1 candidate; G)	Sequence index (pos26)
Arg	tRNA-Arg-TCG-1-1	m2,2G (TRMT1 candidate; G)	Sequence index (pos26)
Arg	tRNA-Arg-TCG-2-1	m2,2G (TRMT1 candidate; G)	Sequence index (pos26)
Arg	tRNA-Arg-TCG-3-1	m2,2G (TRMT1 candidate; G)	Sequence index (pos26)
Arg	tRNA-Arg-TCG-4-1	m2,2G (TRMT1 candidate; G)	Sequence index (pos26)
Arg	tRNA-Arg-TCG-5-1	m2,2G (TRMT1 candidate; G)	Sequence index (pos26)
Arg	tRNA-Arg-TCG-6-1	m2,2G (TRMT1 candidate; G)	Sequence index (pos26)
Leu	tRNA-Leu-CAA-6-1	m2,2G (TRMT1 candidate; G)	Sequence index (pos26)
Leu	tRNA-Leu-TAA-1-1	m2,2G (TRMT1 candidate; G)	Sequence index (pos26)
Leu	tRNA-Leu-TAA-2-1	m2,2G (TRMT1 candidate; G)	Sequence index (pos26)
Leu	tRNA-Leu-TAA-3-1	m2,2G (TRMT1 candidate; G)	Sequence index (pos26)
Leu	tRNA-Leu-TAA-4-1	m2,2G (TRMT1 candidate; G)	Sequence index (pos26)
Lys	tRNA-Lys-CTT-11-1	m2,2G (TRMT1 candidate; G)	Sequence index (pos26)
Met	tRNA-Met-CAT-1-1	m2,2G (TRMT1 candidate; G)	Sequence index (pos26)
Met	tRNA-Met-CAT-2-1	m2,2G (TRMT1 candidate; G)	Sequence index (pos26)
Met	tRNA-Met-CAT-3-1	m2,2G (TRMT1 candidate; G)	Sequence index (pos26)

Met	tRNA-Met-CAT-4-1	m2,2G (TRMT1 candidate; G)	Sequence index (pos26)
Met	tRNA-Met-CAT-5-1	m2,2G (TRMT1 candidate; G)	Sequence index (pos26)
Met	tRNA-Met-CAT-6-1	m2,2G (TRMT1 candidate; G)	Sequence index (pos26)
Met	tRNA-Met-CAT-7-1	m2,2G (TRMT1 candidate; G)	Sequence index (pos26)
Phe	tRNA-Phe-GAA-1-1	m2,2G (TRMT1 candidate; G)	Sequence index (pos26)
Phe	tRNA-Phe-GAA-2-1	m2,2G (TRMT1 candidate; G)	Sequence index (pos26)
Phe	tRNA-Phe-GAA-3-1	m2,2G (TRMT1 candidate; G)	Sequence index (pos26)
Phe	tRNA-Phe-GAA-4-1	m2,2G (TRMT1 candidate; G)	Sequence index (pos26)
Phe	tRNA-Phe-GAA-6-1	m2,2G (TRMT1 candidate; G)	Sequence index (pos26)
SeC	tRNA-SeC-TCA-1-1	m2,2G (TRMT1 candidate; G)	Sequence index (pos26)
Ser	tRNA-Ser-AGA-1-1	m2,2G (TRMT1 candidate; G)	Sequence index (pos26)
Ser	tRNA-Ser-AGA-2-1	m2,2G (TRMT1 candidate; G)	Sequence index (pos26)
Ser	tRNA-Ser-AGA-3-1	m2,2G (TRMT1 candidate; G)	Sequence index (pos26)
Ser	tRNA-Ser-AGA-4-1	m2,2G (TRMT1 candidate; G)	Sequence index (pos26)
Ser	tRNA-Ser-CGA-1-1	m2,2G (TRMT1 candidate; G)	Sequence index (pos26)
Ser	tRNA-Ser-CGA-2-1	m2,2G (TRMT1 candidate; G)	Sequence index (pos26)
Ser	tRNA-Ser-CGA-3-1	m2,2G (TRMT1 candidate; G)	Sequence index (pos26)
Ser	tRNA-Ser-CGA-4-1	m2,2G (TRMT1 candidate; G)	Sequence index (pos26)
Ser	tRNA-Ser-GCT-1-1	m2,2G (TRMT1 candidate; G)	Sequence index (pos26)
Ser	tRNA-Ser-GCT-2-1	m2,2G (TRMT1 candidate; G)	Sequence index (pos26)
Ser	tRNA-Ser-GCT-3-1	m2,2G (TRMT1 candidate; G)	Sequence index (pos26)
Ser	tRNA-Ser-GCT-4-1	m2,2G (TRMT1 candidate; G)	Sequence index (pos26)
Ser	tRNA-Ser-GCT-5-1	m2,2G (TRMT1 candidate; G)	Sequence index (pos26)
Ser	tRNA-Ser-GCT-6-1	m2,2G (TRMT1 candidate; G)	Sequence index (pos26)
Ser	tRNA-Ser-TGA-1-1	m2,2G (TRMT1 candidate; G)	Sequence index (pos26)
Ser	tRNA-Ser-TGA-2-1	m2,2G (TRMT1 candidate; G)	Sequence index (pos26)
Ser	tRNA-Ser-TGA-3-1	m2,2G (TRMT1 candidate; G)	Sequence index (pos26)
Ser	tRNA-Ser-TGA-4-1	m2,2G (TRMT1 candidate; G)	Sequence index (pos26)
Thr	tRNA-Thr-CGT-5-1	m2,2G (TRMT1 candidate; G)	Sequence index (pos26)
Thr	tRNA-Thr-TGT-3-1	m2,2G (TRMT1 candidate; G)	Sequence index (pos26)
Tyr	tRNA-Tyr-GTA-1-1	m2,2G (TRMT1 candidate; G)	Sequence index (pos26)
Tyr	tRNA-Tyr-GTA-2-1	m2,2G (TRMT1 candidate; G)	Sequence index (pos26)
Tyr	tRNA-Tyr-GTA-3-1	m2,2G (TRMT1 candidate; G)	Sequence index (pos26)
Tyr	tRNA-Tyr-GTA-4-1	m2,2G (TRMT1 candidate; G)	Sequence index (pos26)
Tyr	tRNA-Tyr-GTA-5-1	m2,2G (TRMT1 candidate; G)	Sequence index (pos26)
Tyr	tRNA-Tyr-GTA-6-1	m2,2G (TRMT1 candidate; G)	Sequence index (pos26)
Tyr	tRNA-Tyr-GTA-7-1	m2,2G (TRMT1 candidate; G)	Sequence index (pos26)
Tyr	tRNA-Tyr-GTA-8-1	m2,2G (TRMT1 candidate; G)	Sequence index (pos26)
Tyr	tRNA-Tyr-GTA-9-1	m2,2G (TRMT1 candidate; G)	Sequence index (pos26)
Val	tRNA-Val-AAC-1-1	m2G (TRMT1-dependent; Val/iMet)	Isotype rule (Val/iMet)
Val	tRNA-Val-AAC-2-1	m2G (TRMT1-dependent; Val/iMet)	Isotype rule (Val/iMet)
Val	tRNA-Val-AAC-3-1	m2G (TRMT1-dependent; Val/iMet)	Isotype rule (Val/iMet)
Val	tRNA-Val-AAC-4-1	m2G (TRMT1-dependent; Val/iMet)	Isotype rule (Val/iMet)
Val	tRNA-Val-AAC-5-1	m2G (TRMT1-dependent; Val/iMet)	Isotype rule (Val/iMet)
Val	tRNA-Val-CAC-1-1	m2G (TRMT1-dependent; Val/iMet)	Isotype rule (Val/iMet)
Val	tRNA-Val-CAC-2-1	m2G (TRMT1-dependent; Val/iMet)	Isotype rule (Val/iMet)

Val	tRNA-Val-CAC-4-1	m2G (TRMT1-dependent; Val/iMet)	Isotype rule (Val/iMet)
Val	tRNA-Val-CAC-6-1	m2G (TRMT1-dependent; Val/iMet)	Isotype rule (Val/iMet)
Val	tRNA-Val-TAC-1-1	m2G (TRMT1-dependent; Val/iMet)	Isotype rule (Val/iMet)
Val	tRNA-Val-TAC-2-1	m2G (TRMT1-dependent; Val/iMet)	Isotype rule (Val/iMet)
iMet	tRNA-iMet-CAT-1-1	m2G (TRMT1-dependent; Val/iMet)	Isotype rule (Val/iMet)
iMet	tRNA-iMet-CAT-2-1	m2G (TRMT1-dependent; Val/iMet)	Isotype rule (Val/iMet)

Predicted m2,2G or m2G modifications at position 26 were assigned according to the nucleotide identity at position 26 in the mature tRNA sequence or according to isotype-specific rules for Val/iMet tRNAs. "Sequence index (pos26)" indicates that G26 was identified by mature tRNA sequence indexing. "TRMT1 candidate" refers to putative TRMT1 substrates predicted from the presence of G26, while "TRMT1-dependent" indicates modification assignment based on known Val/iMet isotype rules. These annotations represent computational predictions and require experimental validation.
