# Supplementary material for: TRMT1-mediated tRNA m22G modification drives Osimertinib resistance via the ATXN3/USP25 axis in lung adenocarcinoma
Source: Cell Death Dis. 2026 Jun 29;17(1):660. doi: 10.1038/s41419-026-09039-8 (PMC13408171; doi:10.1038/s41419-026-09039-8)

Silver-stained gel

ATXN3 MS

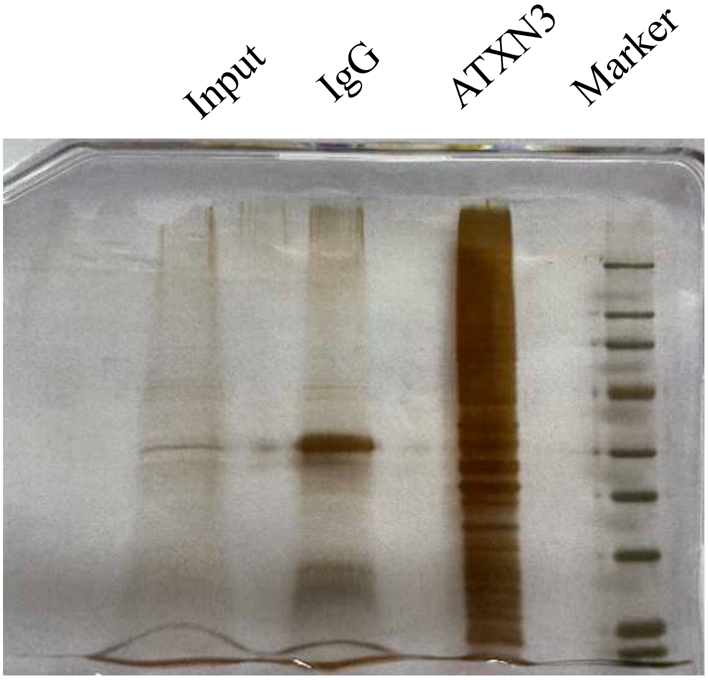

USP25 MS

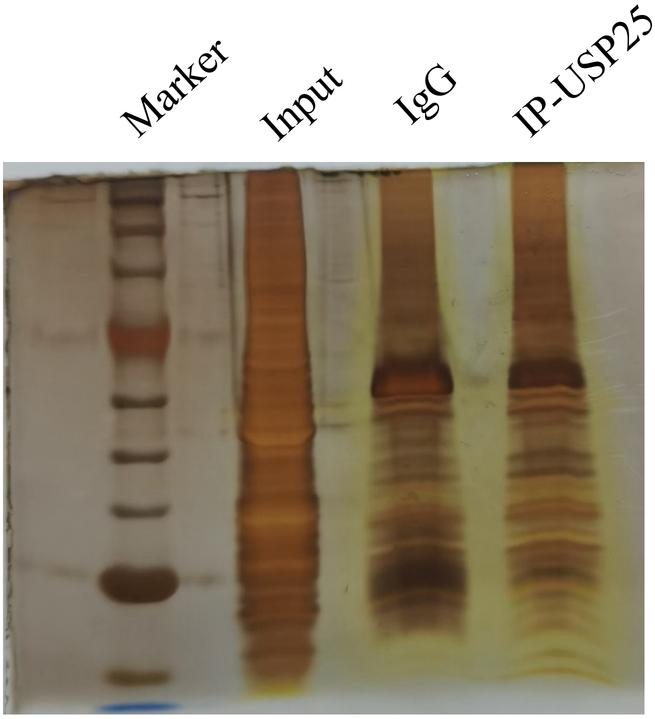

1A

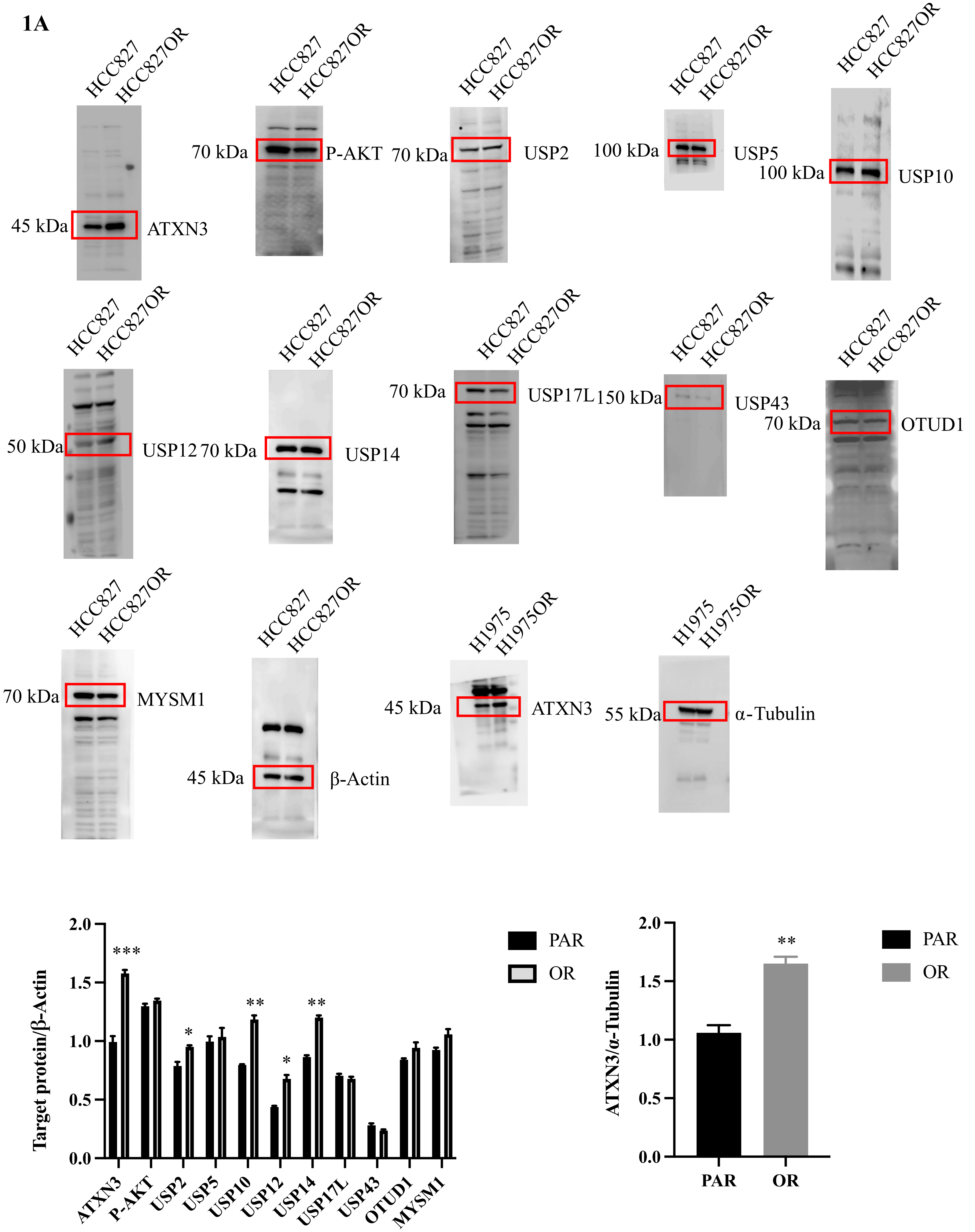
HCC827
HCC827OR
45 kDa
ATXN3
70 kDa
P-AKT
70 kDa
USP2
100 kDa
USP5
100 kDa
USP10
50 kDa
USP12
70 kDa
USP14
70 kDa
USP17L
150 kDa
USP43
70 kDa
OTUD1
70 kDa
MYSM1
45 kDa
β-Actin
H1975
H1975OR
45 kDa
ATXN3
55 kDa
α-Tubulin
Target protein/β-Actin
0.0
0.5
1.0
1.5
2.0
***
*
**
*
**
ATXN3
P-AKT
USP2
USP5
USP10
USP12
USP14
USP17L
USP43
OTUD1
MYSM1
PAR
OR
ATXN3/α-Tubulin
**
PAR
OR

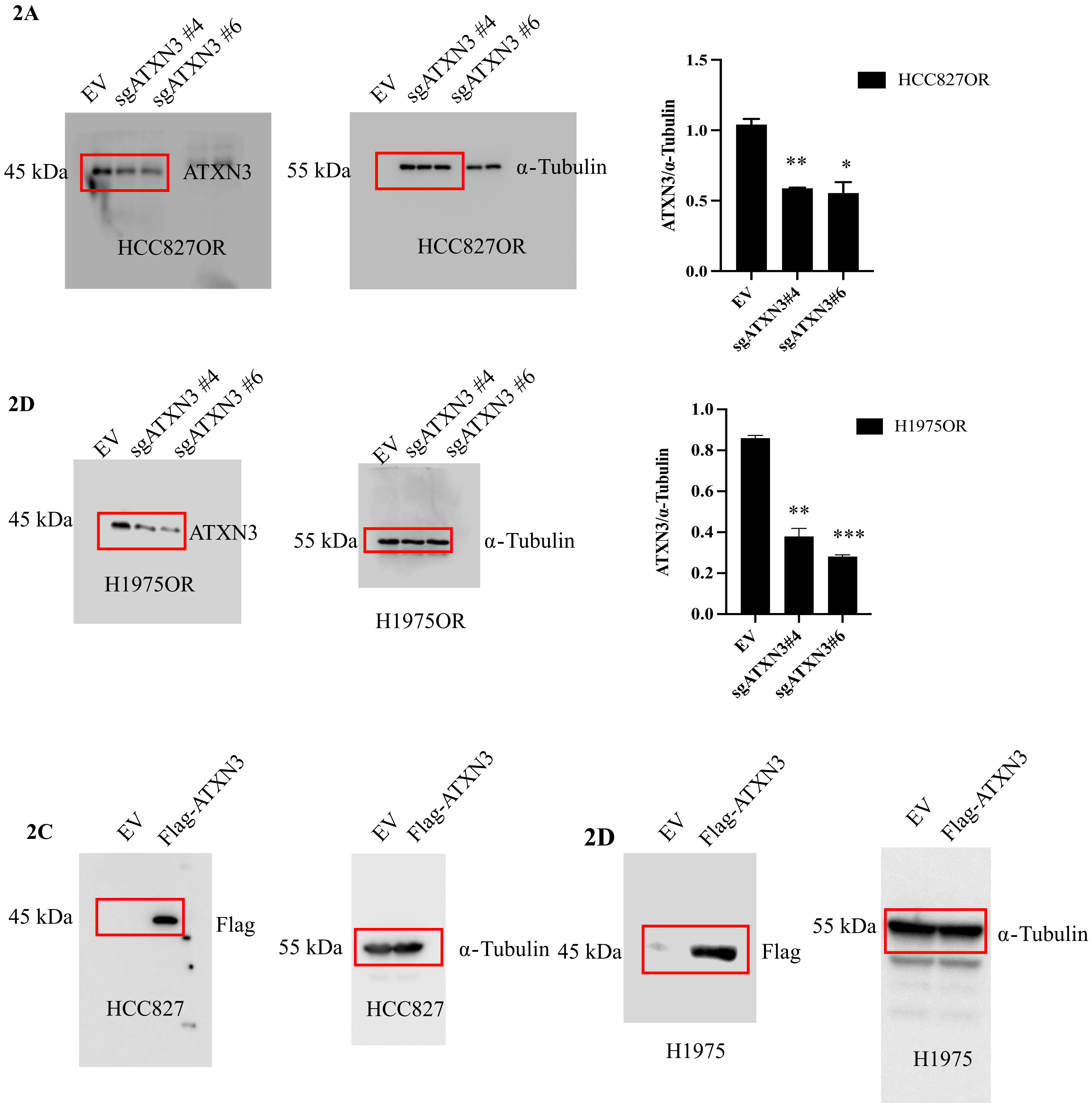
2A
EV
sgATXN3 #4
sgATXN3 #6
45 kDa
ATXN3
HCC827OR
55 kDa
α-Tubulin
HCC827OR
ATXN3/α-Tubulin
1.5
1.0
0.5
0.0
HCC827OR
**
*
EV
sgATXN3#4
sgATXN3#6
2D
EV
sgATXN3 #4
sgATXN3 #6
45 kDa
ATXN3
H1975OR
55 kDa
α-Tubulin
H1975OR
ATXN3/α-Tubulin
1.0
0.8
0.6
0.4
0.2
0.0
H1975OR
**
***
EV
sgATXN3#4
sgATXN3#6
2C
EV
Flag-ATXN3
45 kDa
Flag
HCC827
55 kDa
α-Tubulin
HCC827
2D
EV
Flag-ATXN3
45 kDa
Flag
H1975
55 kDa
α-Tubulin
H1975

3B

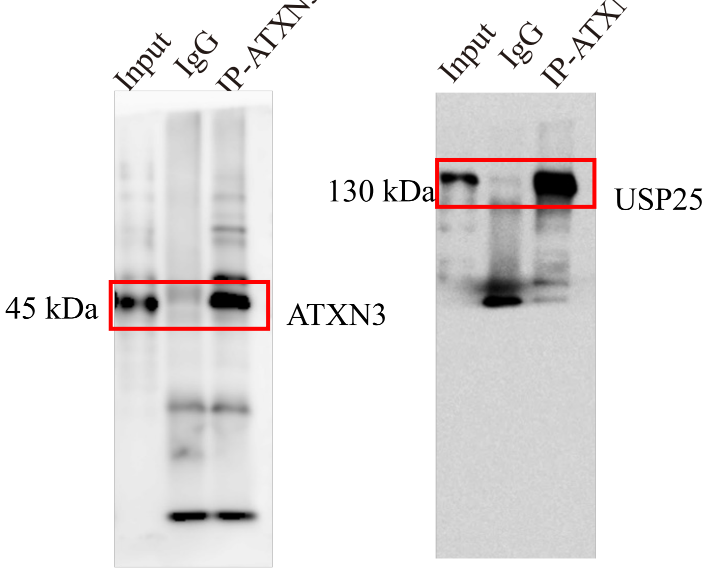

Input
IgG
IP-ATXN3
45 kDa
ATXN3
Input
IgG
IP-ATXN3
130 kDa
USP25

3C

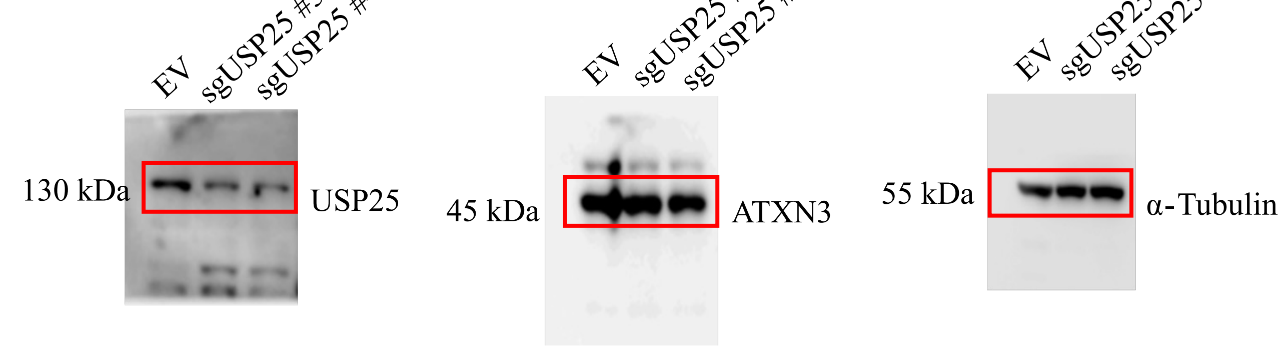

EV
sgUSP25 #3
sgUSP25 #4
130 kDa
USP25
EV
sgUSP25 #3
sgUSP25 #4
45 kDa
ATXN3
EV
sgUSP25 #3
sgUSP25 #4
55 kDa
α-Tubulin

3D

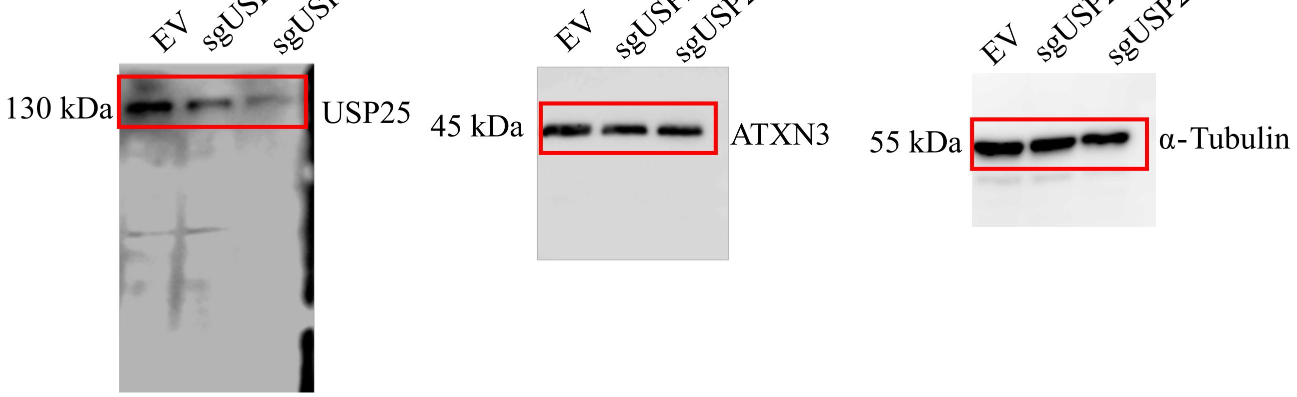

EV
sgUSP25 #3
sgUSP25 #4
130 kDa
USP25
EV
sgUSP25 #3
sgUSP25 #4
45 kDa
ATXN3
EV
sgUSP25 #3
sgUSP25 #4
55 kDa
α-Tubulin

3C

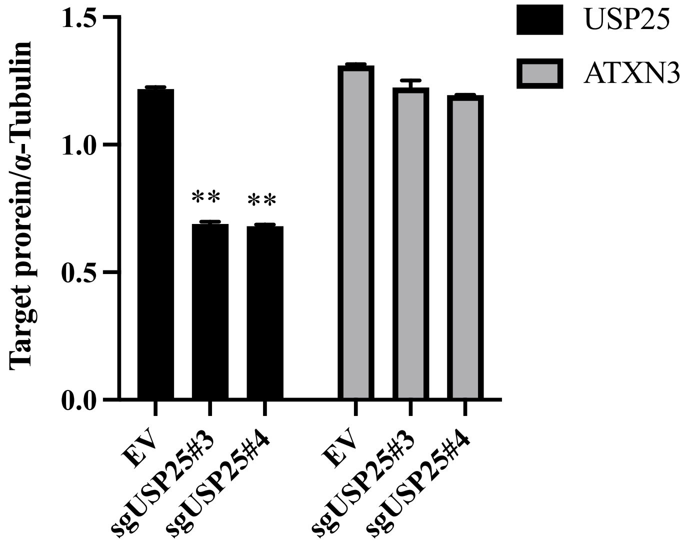

Target prorein/α-Tubulin
1.5
1.0
0.5
0.0
USP25
ATXN3
**
**
EV
sgUSP25#3
sgUSP25#4
EV
sgUSP25#3
sgUSP25#4

3D

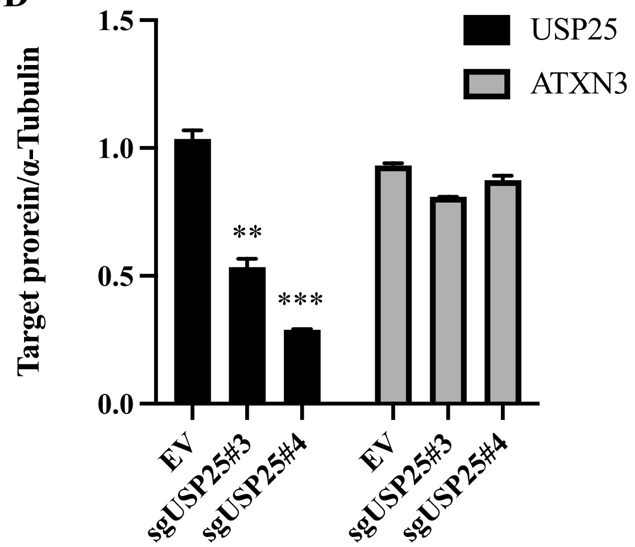

Target prorein/α-Tubulin
1.5
1.0
0.5
0.0
USP25
ATXN3
**
***
EV
sgUSP25#3
sgUSP25#4
EV
sgUSP25#3
sgUSP25#4

4A

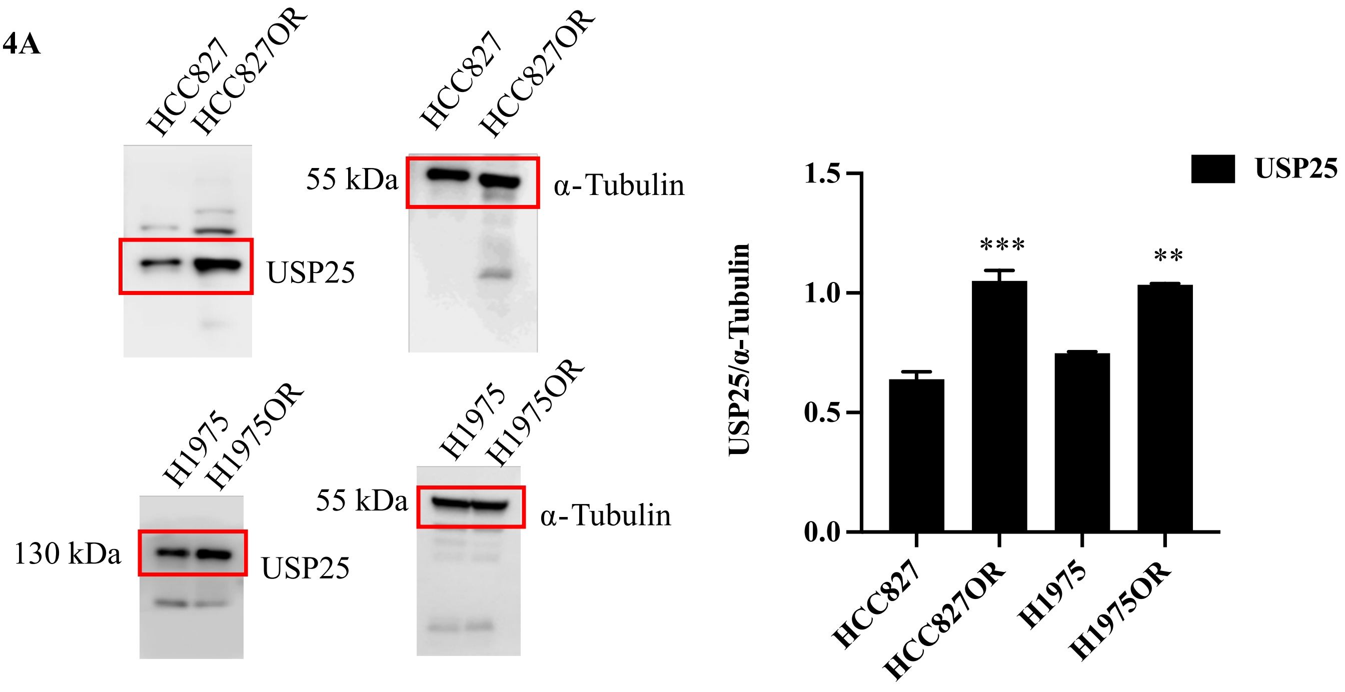
HCC827
HCC827OR
USP25
55 kDa
α-Tubulin
H1975
H1975OR
130 kDa
USP25
55 kDa
α-Tubulin
USP25
USP25/α-Tubulin
1.5
1.0
0.5
0.0
***
**
HCC827
HCC827OR
H1975
H1975OR

4B

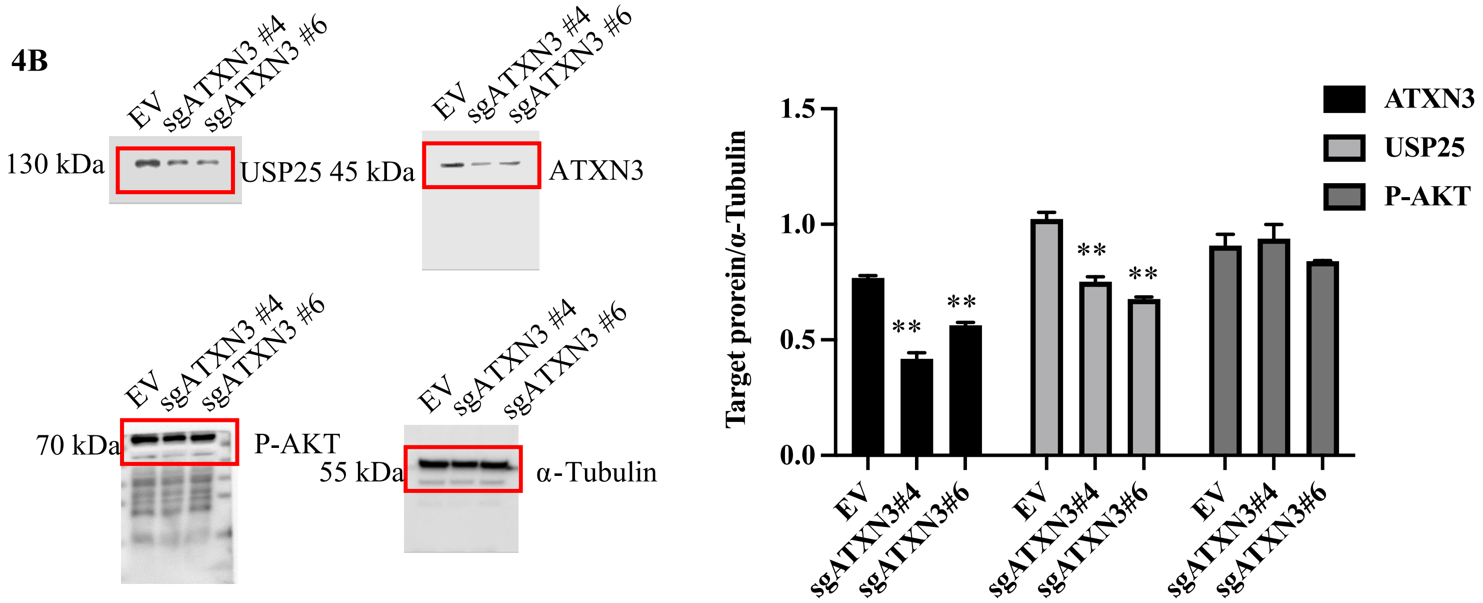
EV
sgATXN3 #4
sgATXN3 #6
130 kDa
USP25
45 kDa
ATXN3
70 kDa
P-AKT
55 kDa
α-Tubulin
ATXN3
USP25
P-AKT
Target prorein/α-Tubulin
1.5
1.0
0.5
0.0
**
EV
sgATXN3#4
sgATXN3#6

4C

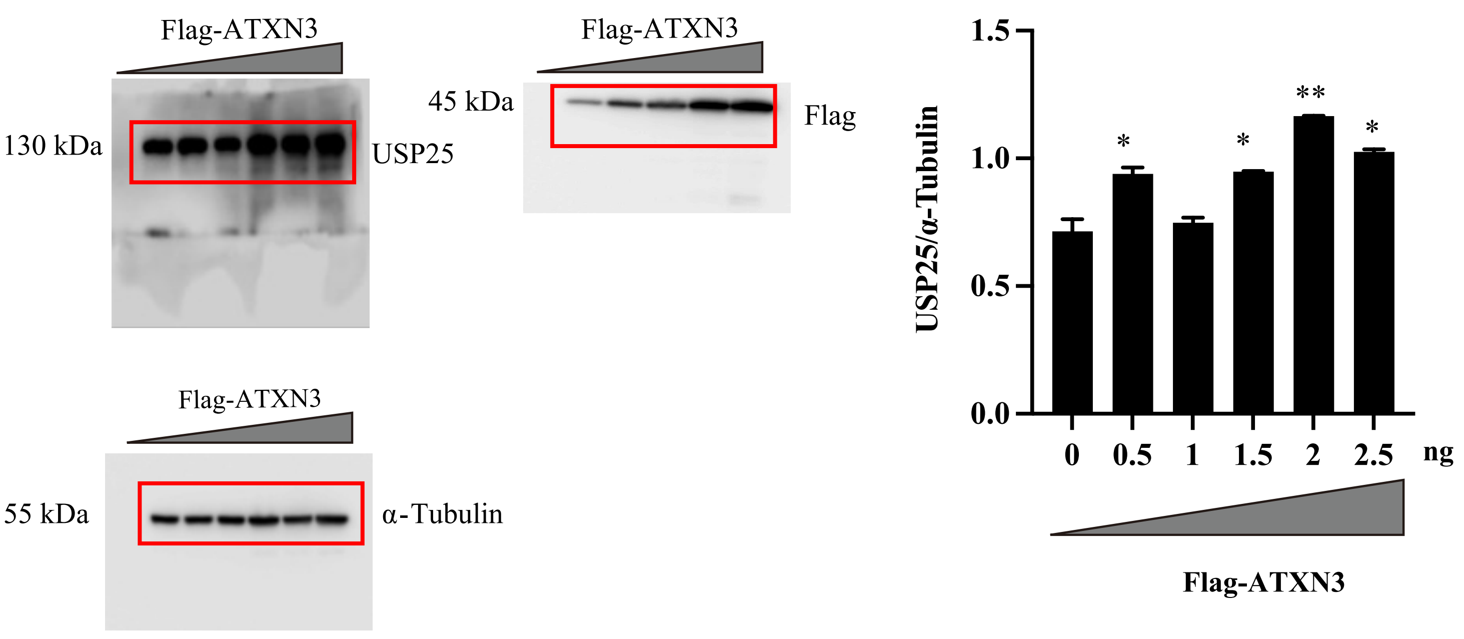
Flag-ATXN3
130 kDa
USP25
45 kDa
Flag
55 kDa
α-Tubulin
USP25/α-Tubulin
1.5
1.0
0.5
0.0
*
**
0
0.5
1
1.5
2
2.5
ng
Flag-ATXN3

4D

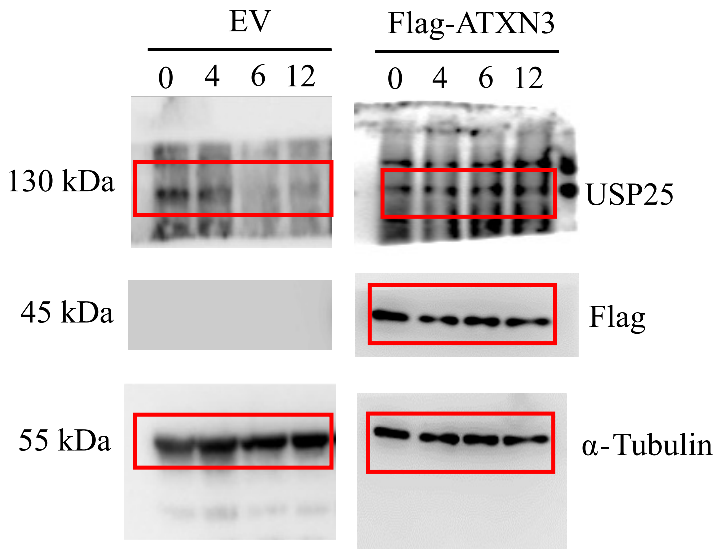

EV
Flag-ATXN3
0 4 6 12
0 4 6 12
130 kDa
45 kDa
55 kDa
USP25
Flag
α-Tubulin

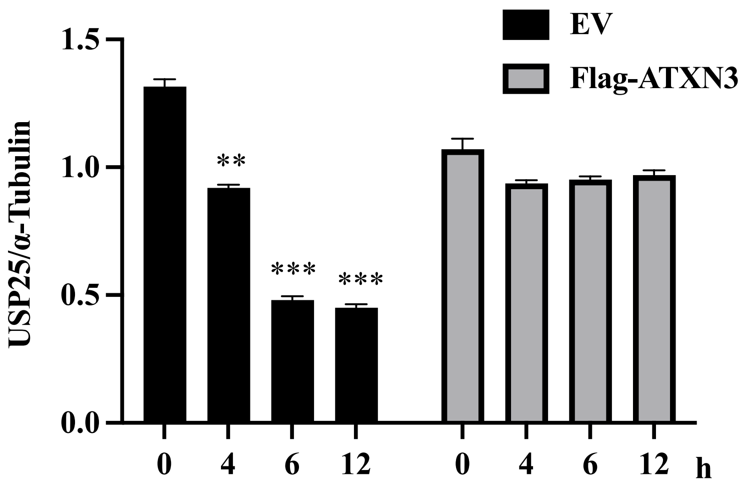

EV
Flag-ATXN3
USP25/α-Tubulin
1.5
1.0
0.5
0.0
**
***
***
0 4 6 12 0 4 6 12 h

4E

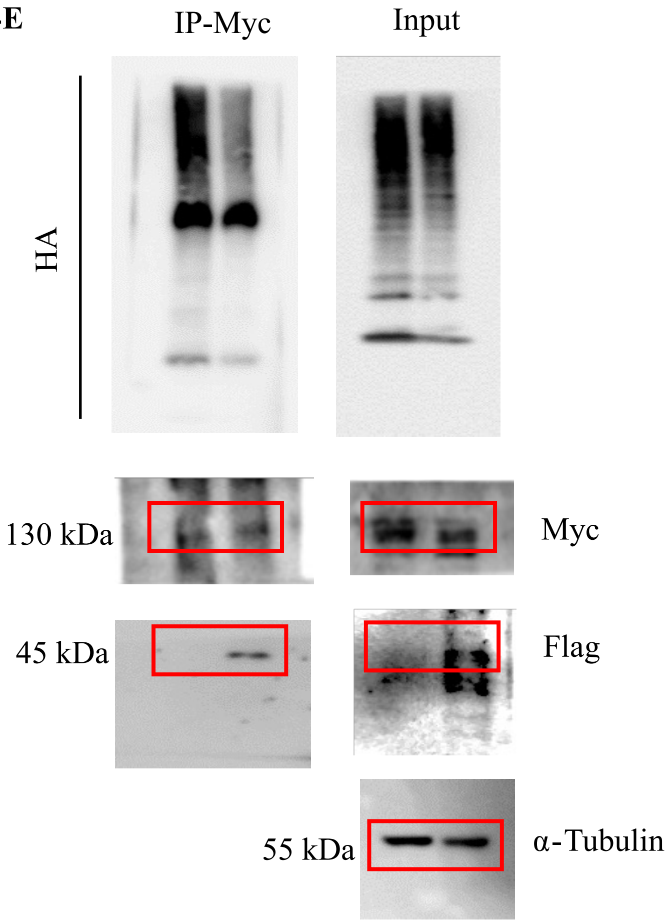

IP-Myc
Input
HA
130 kDa
45 kDa
55 kDa
Myc
Flag
α-Tubulin

4F

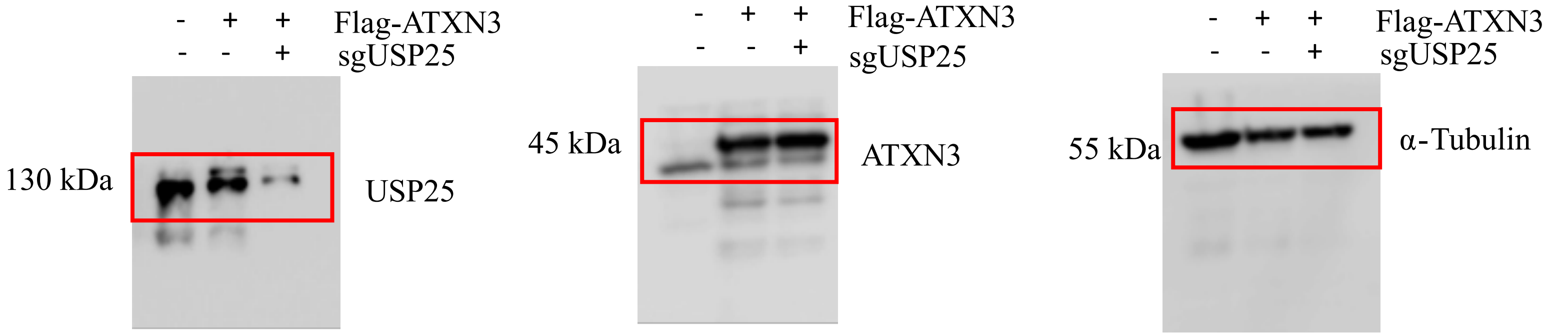
- + + Flag-ATXN3
- - + sgUSP25
130 kDa
USP25
45 kDa
ATXN3
55 kDa
α-Tubulin

4G

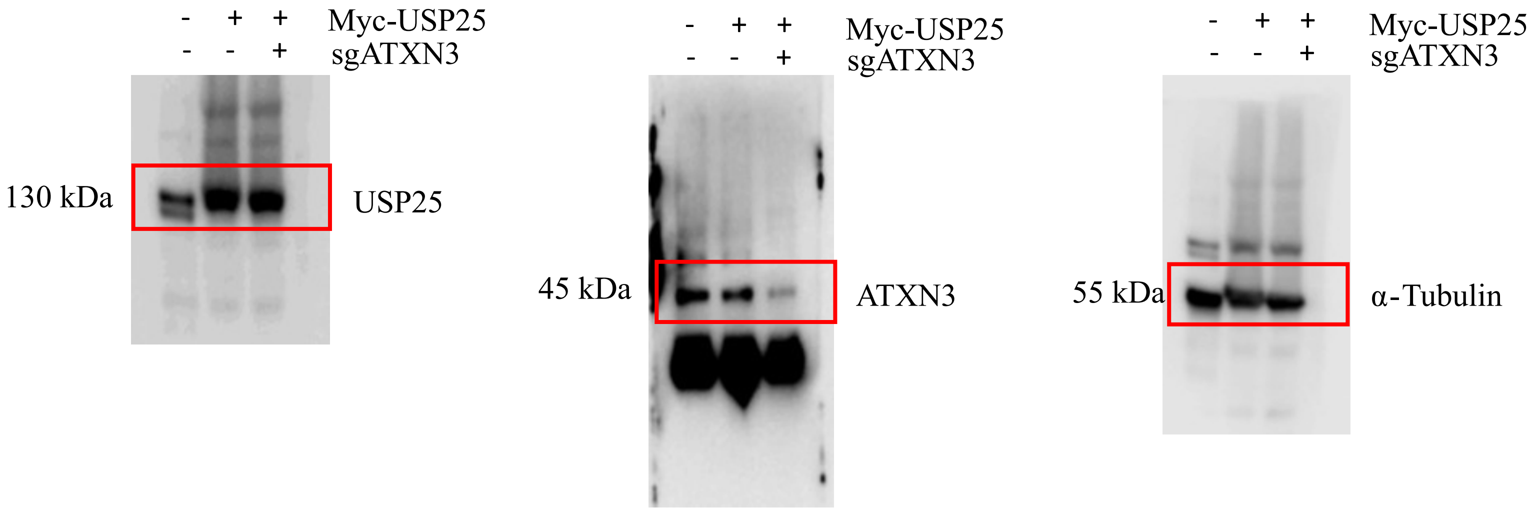
- + + Myc-USP25
- - + sgATXN3
130 kDa
USP25
45 kDa
ATXN3
55 kDa
α-Tubulin

4H

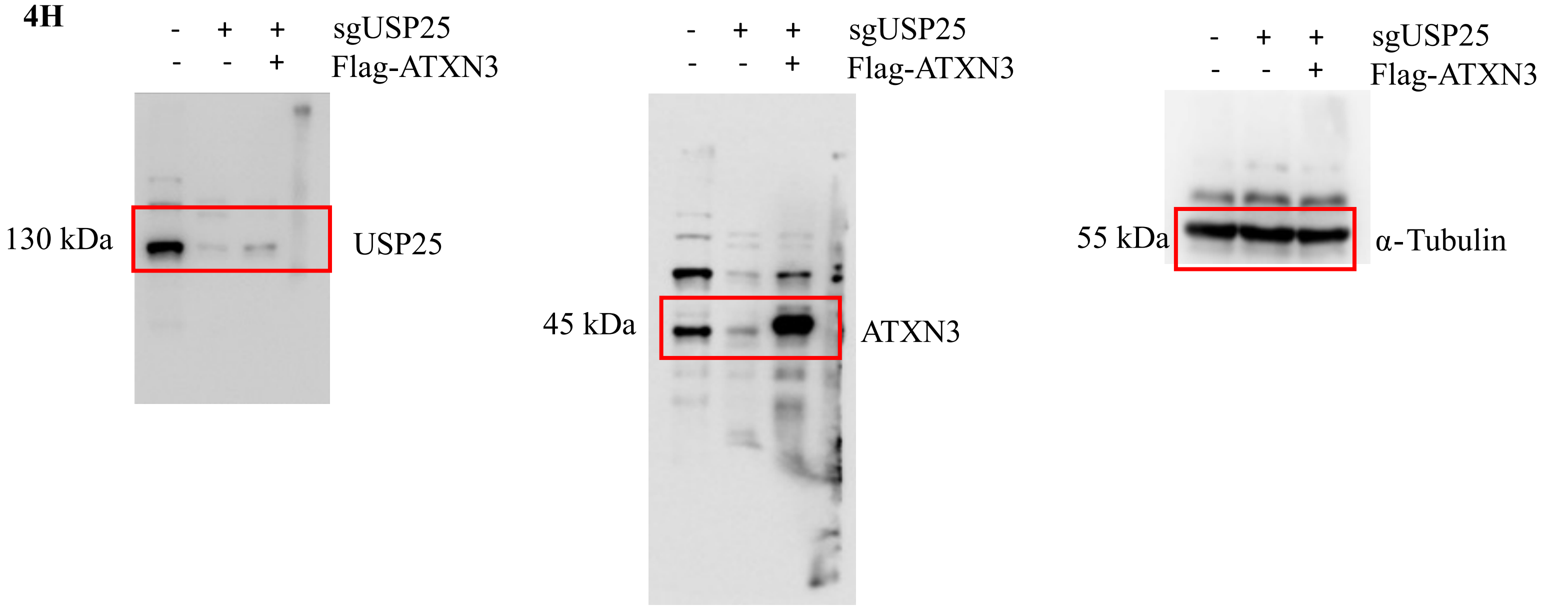
- + + sgUSP25
- - + Flag-ATXN3
130 kDa
USP25
45 kDa
ATXN3
55 kDa
α-Tubulin

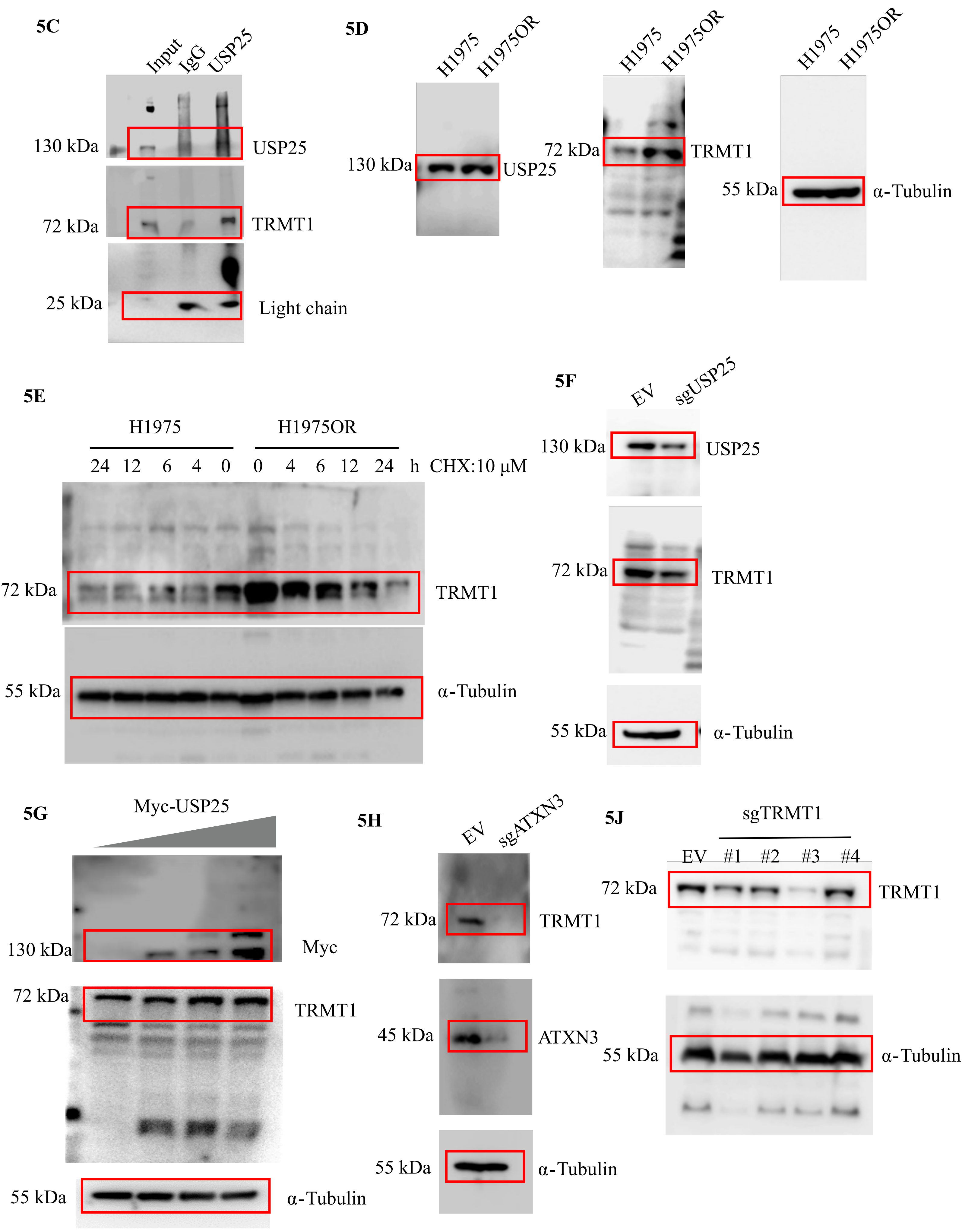
5C
Input
IgG
USP25
130 kDa
USP25
72 kDa
TRMT1
25 kDa
Light chain
5D
H1975
H1975OR
130 kDa
USP25
H1975
H1975OR
72 kDa
TRMT1
H1975
H1975OR
55 kDa
α-Tubulin
5E
H1975
H1975OR
24 12 6 4 0 0 4 6 12 24 h CHX:10 μM
72 kDa
TRMT1
55 kDa
α-Tubulin
5F
EV
sgUSP25
130 kDa
USP25
72 kDa
TRMT1
55 kDa
α-Tubulin
5G
Myc-USP25
130 kDa
Myc
72 kDa
TRMT1
55 kDa
α-Tubulin
5H
EV
sgATXN3
72 kDa
TRMT1
45 kDa
ATXN3
55 kDa
α-Tubulin
5J
sgTRMT1
EV #1 #2 #3 #4
72 kDa
TRMT1
55 kDa
α-Tubulin

5D

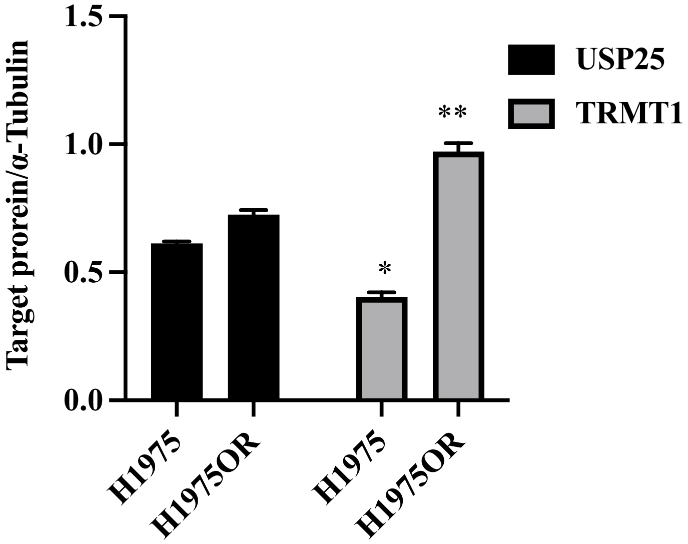

5E

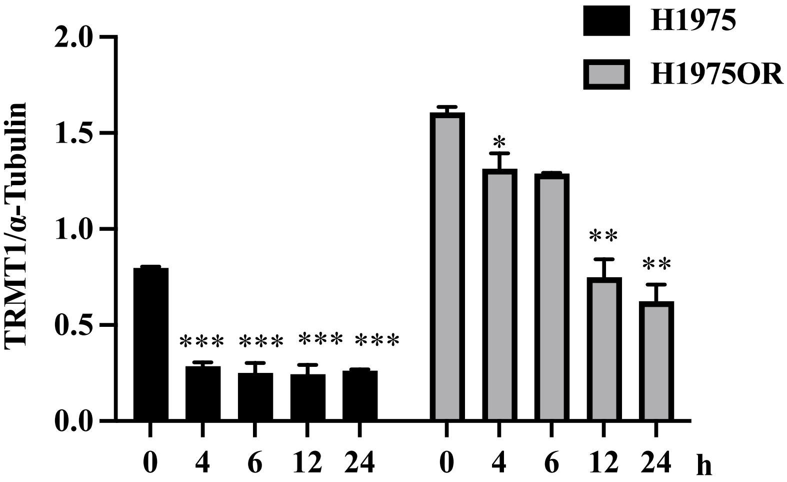

5F

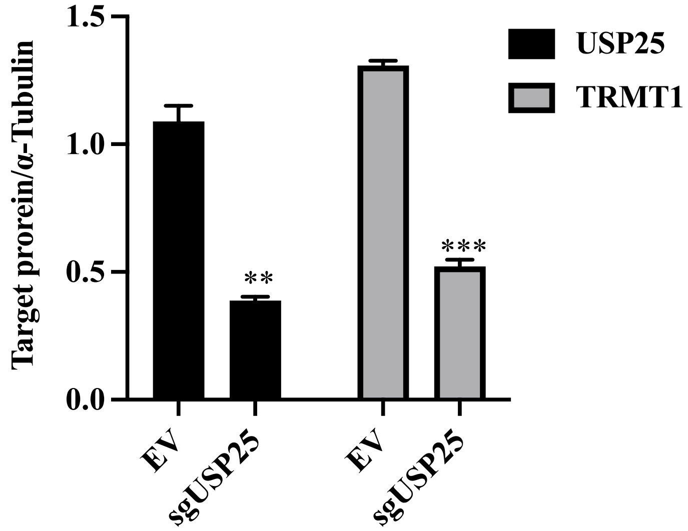

5G

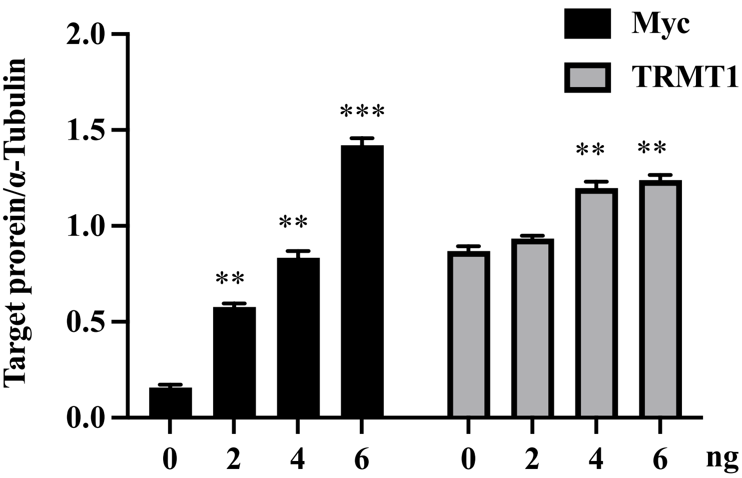

5H

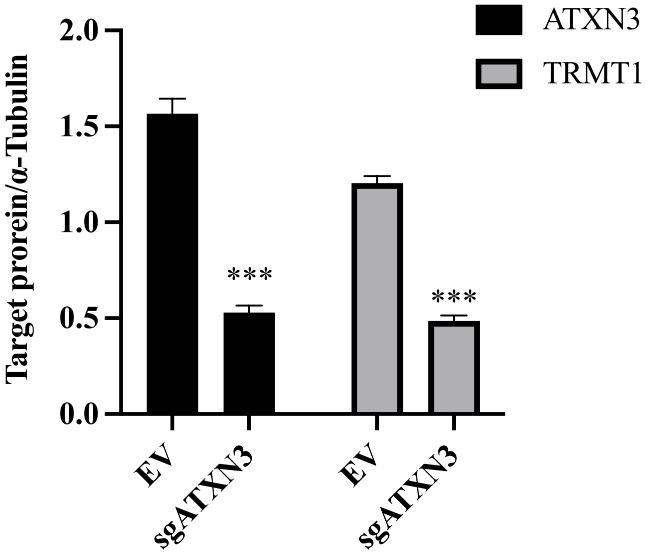

5J

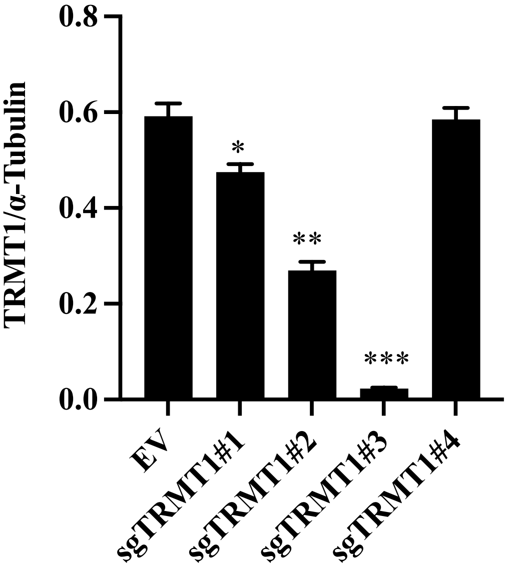

6E

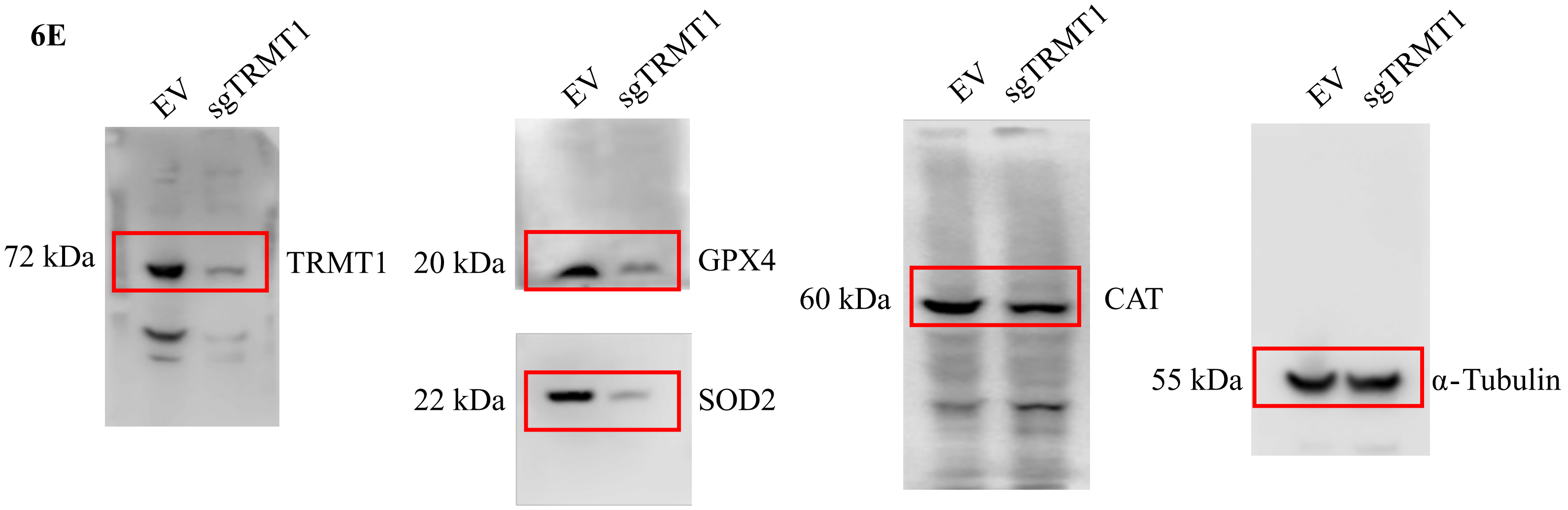
EV
sgTRMT1
72 kDa
TRMT1
20 kDa
GPX4
22 kDa
SOD2
60 kDa
CAT
55 kDa
α-Tubulin

6F

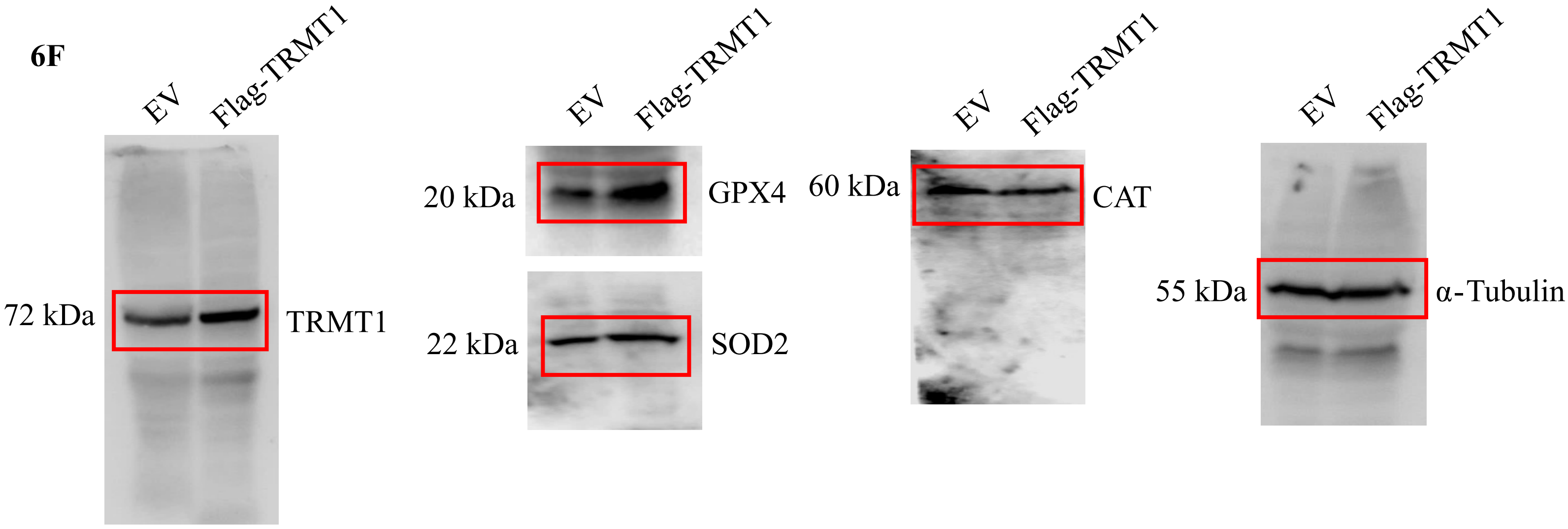
EV
Flag-TRMT1
72 kDa
TRMT1
20 kDa
GPX4
22 kDa
SOD2
60 kDa
CAT
55 kDa
α-Tubulin

6H

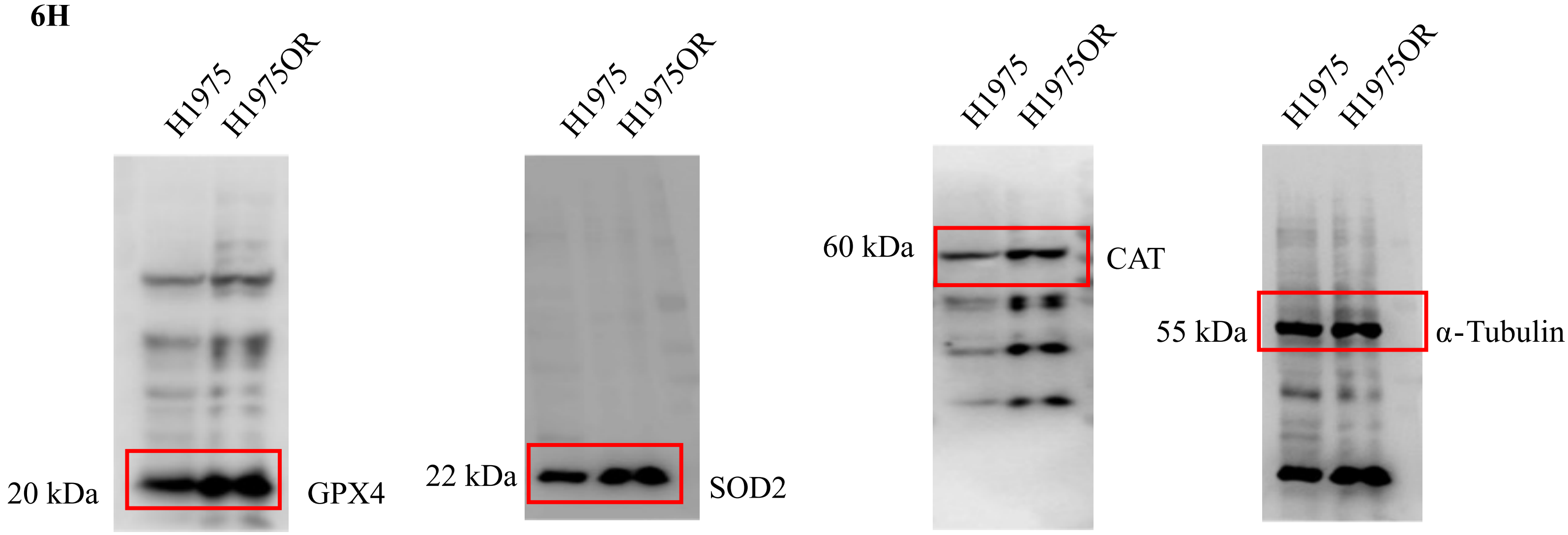
H1975
H1975OR
20 kDa
GPX4
22 kDa
SOD2
60 kDa
CAT
55 kDa
α-Tubulin

6E

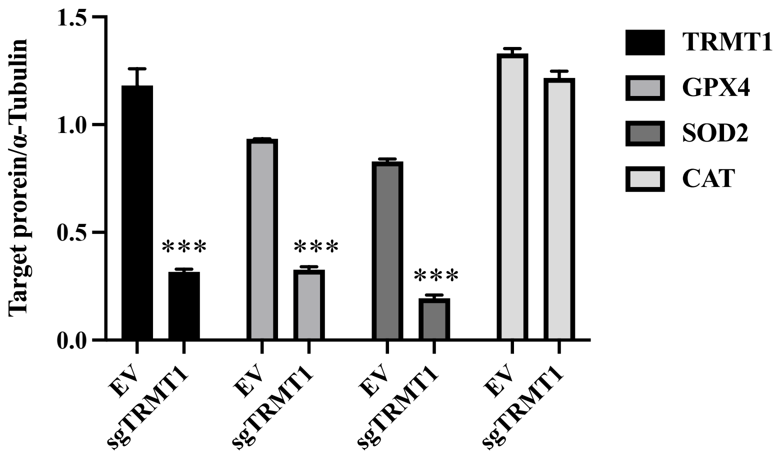

6F

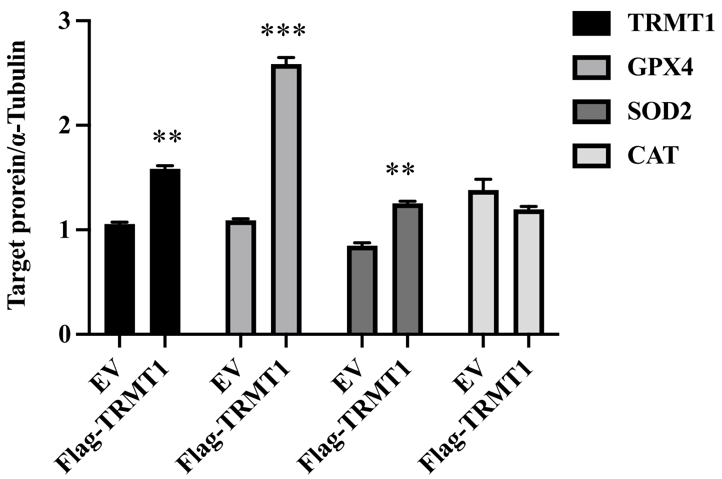

6G

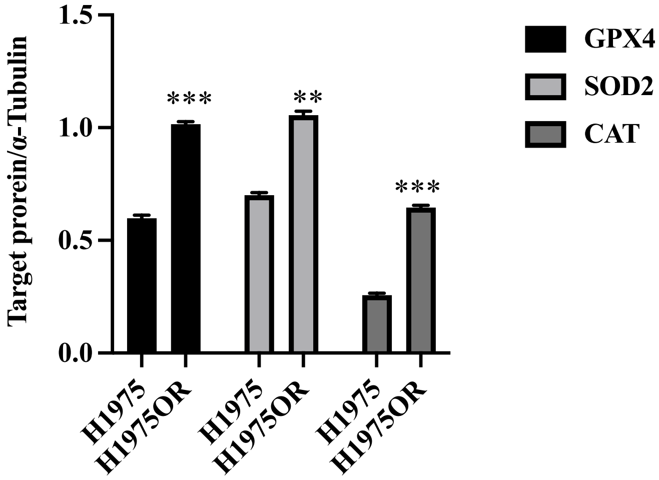

S2A

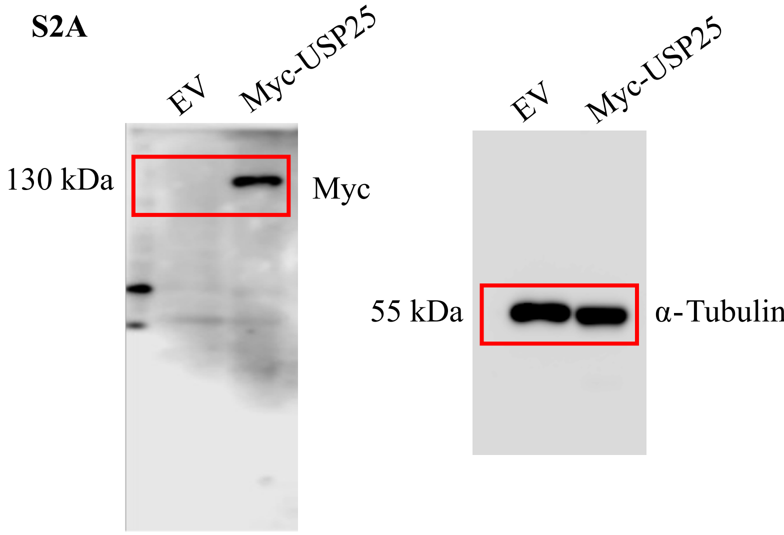

S2D

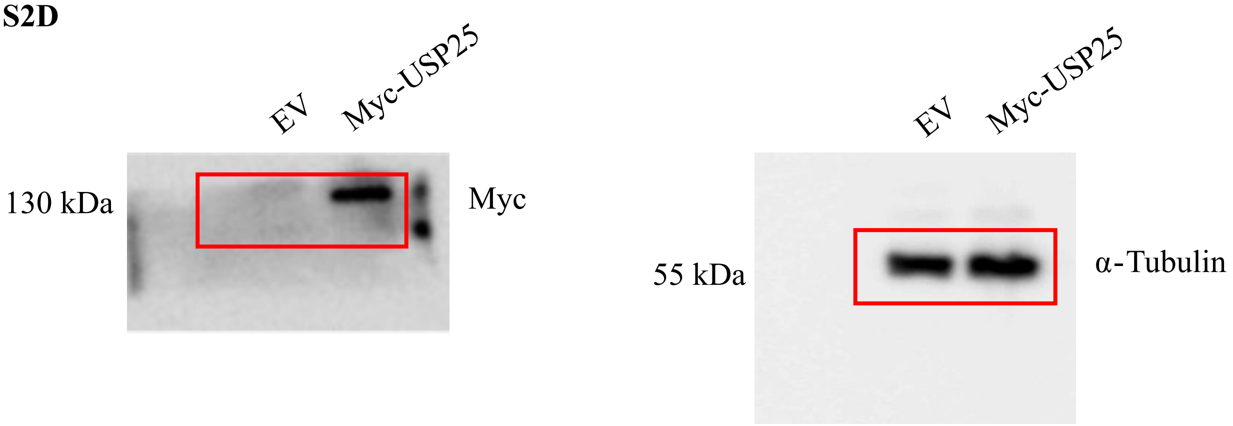

S3A

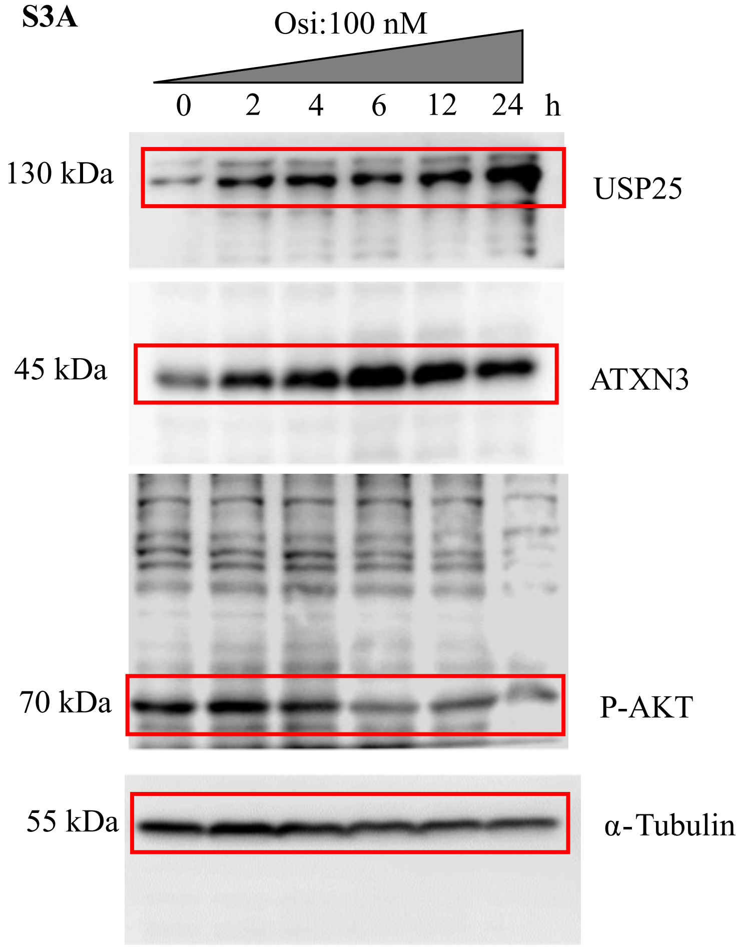

S3B

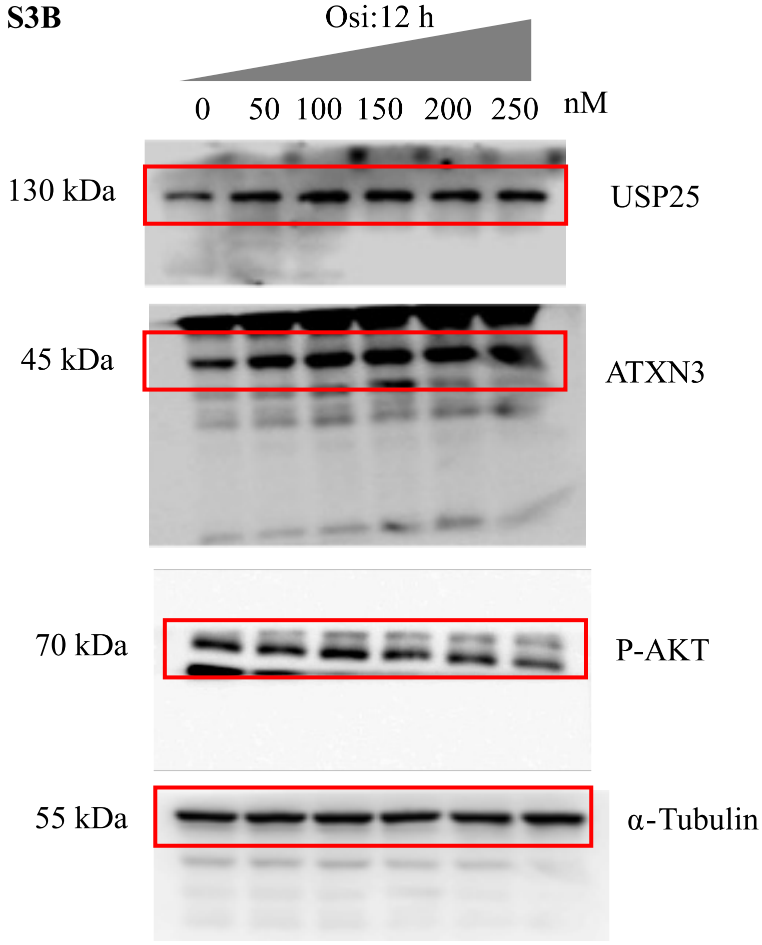

S3C

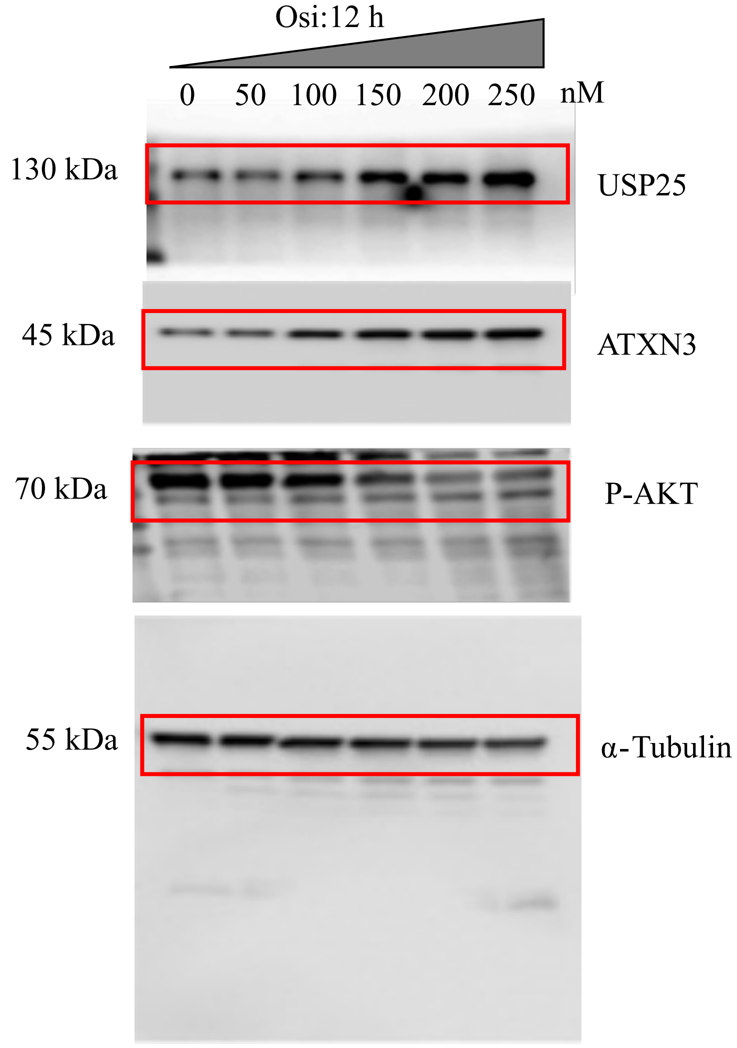

S3E

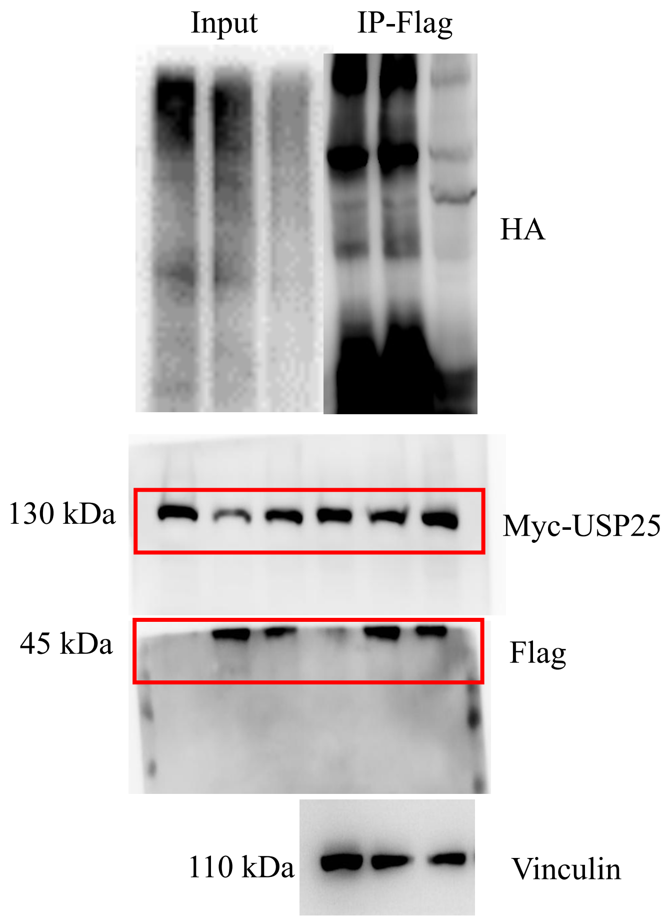

S4A

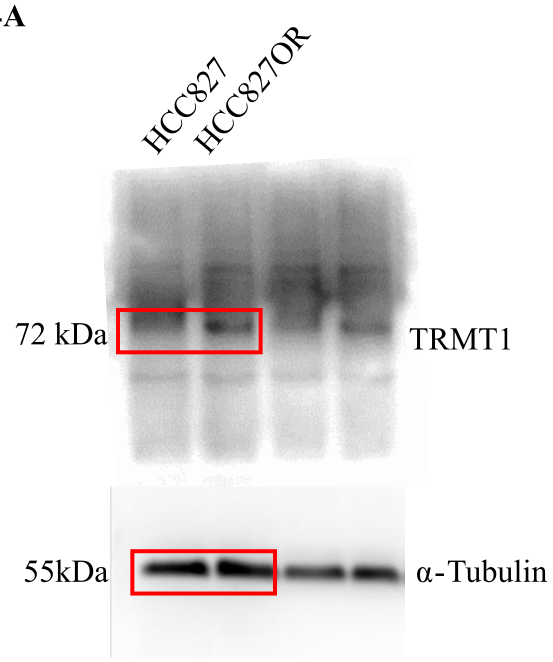

S4B

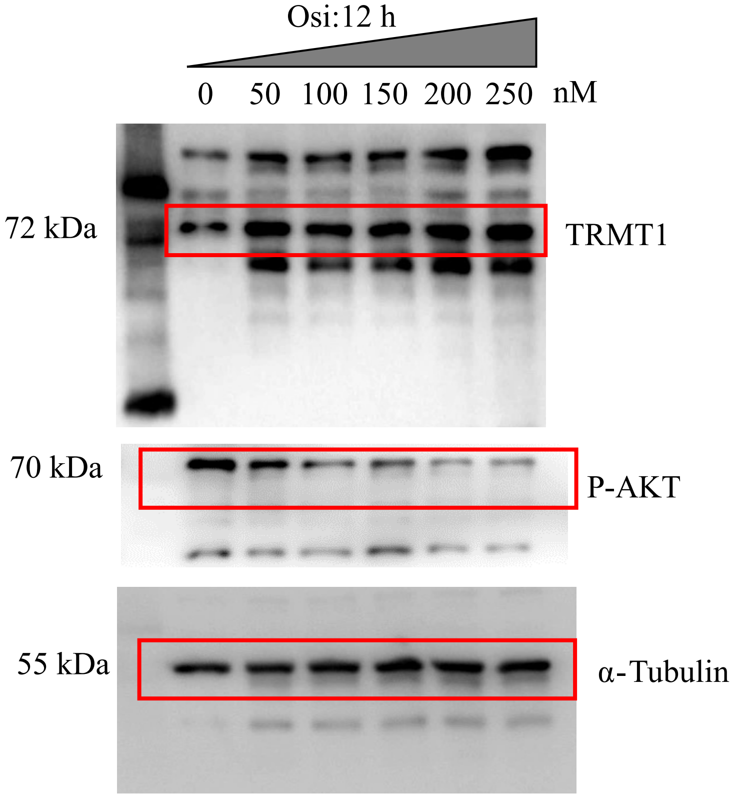

S4C

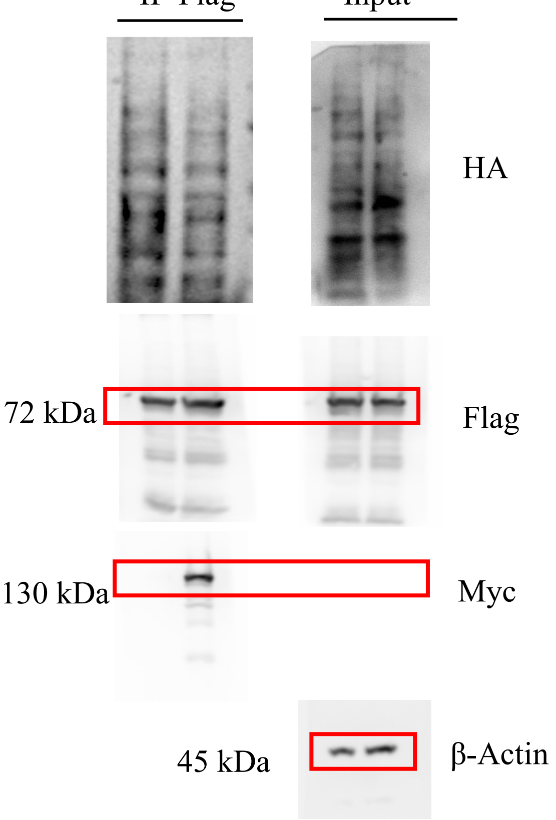

S4D

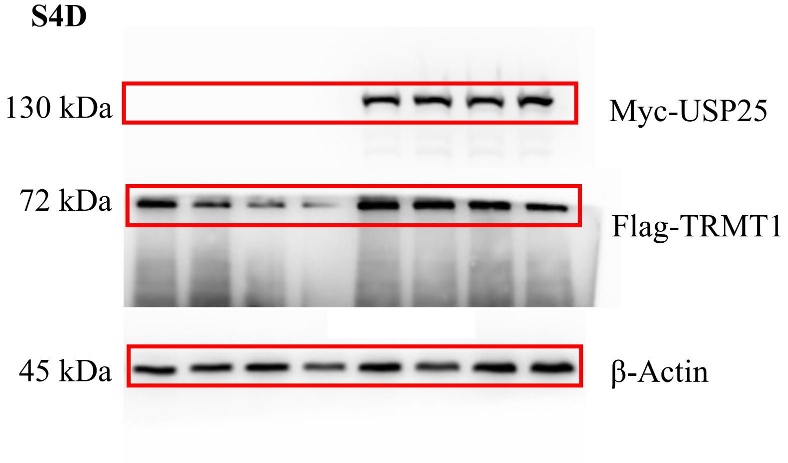

S4E

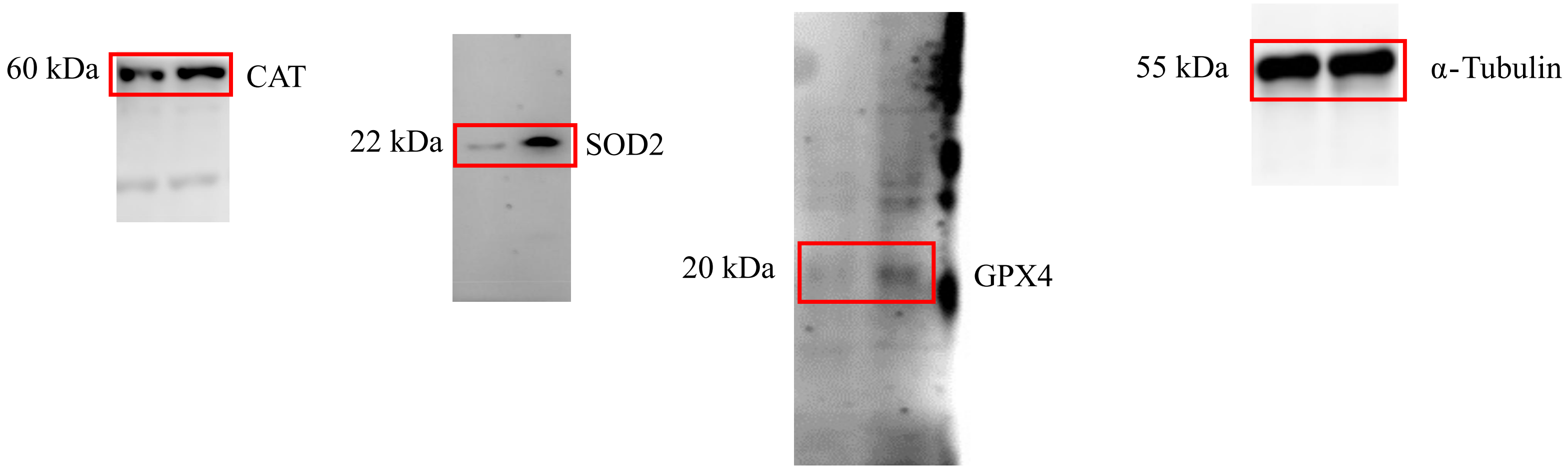

S3A

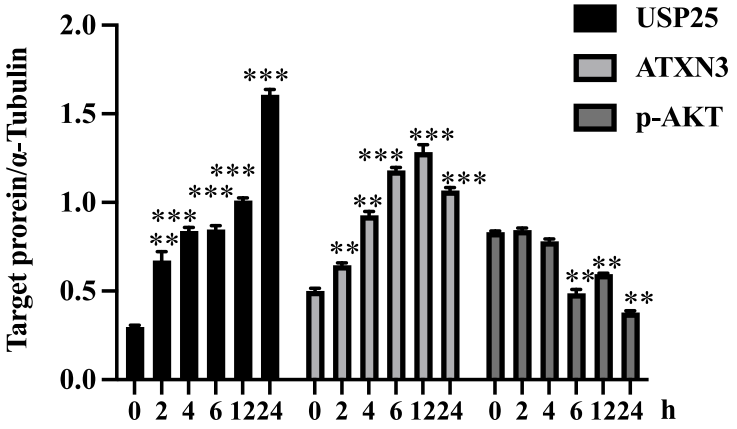

S3B

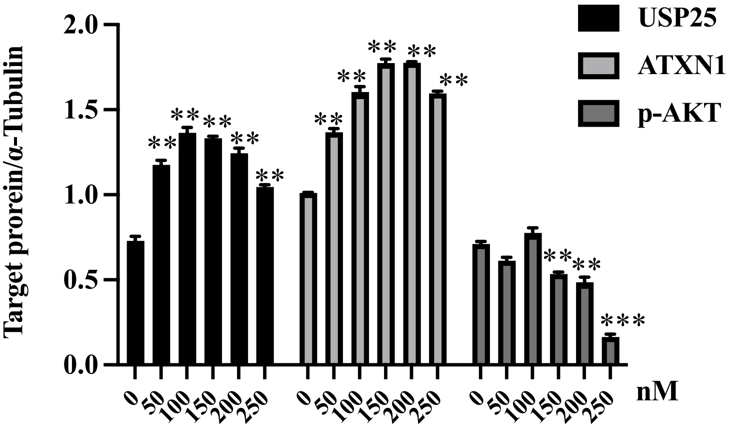

S3C

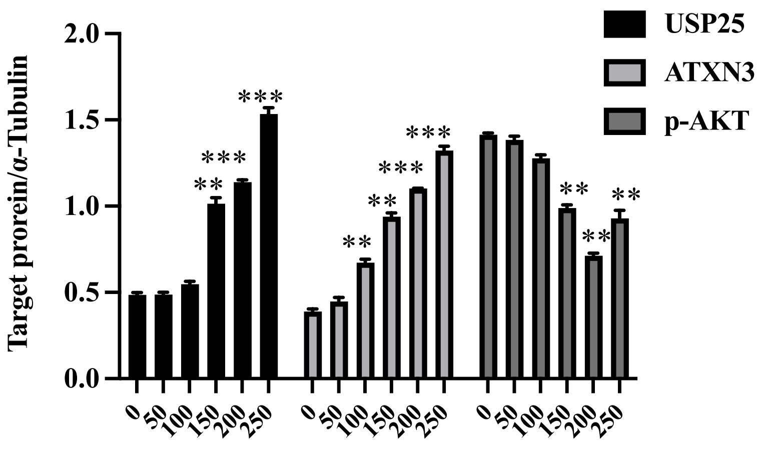

S4A

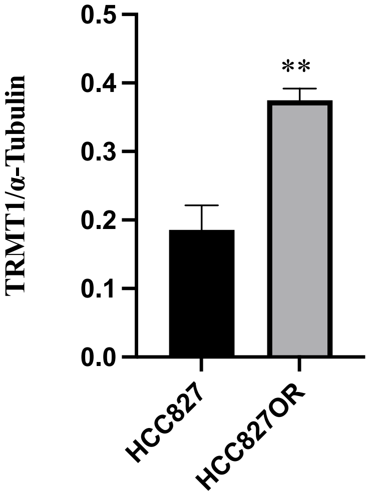

S4B

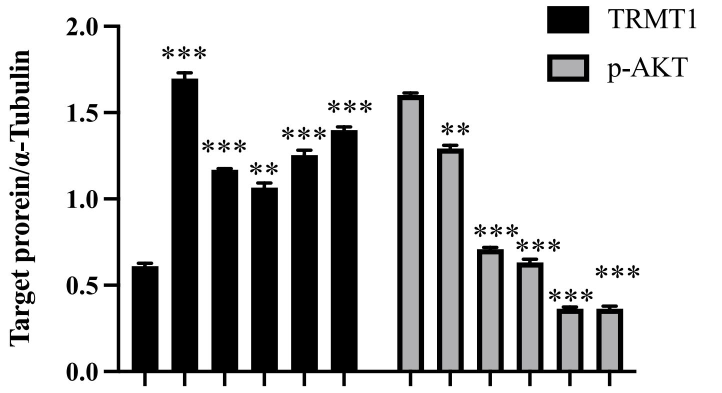

S4D

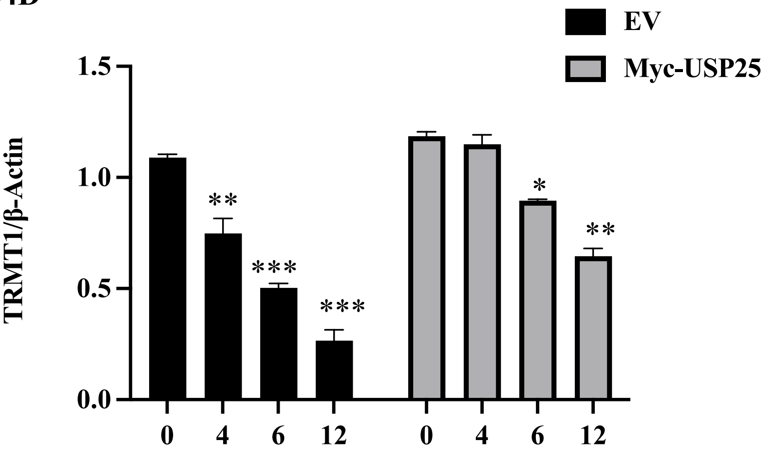

S4E

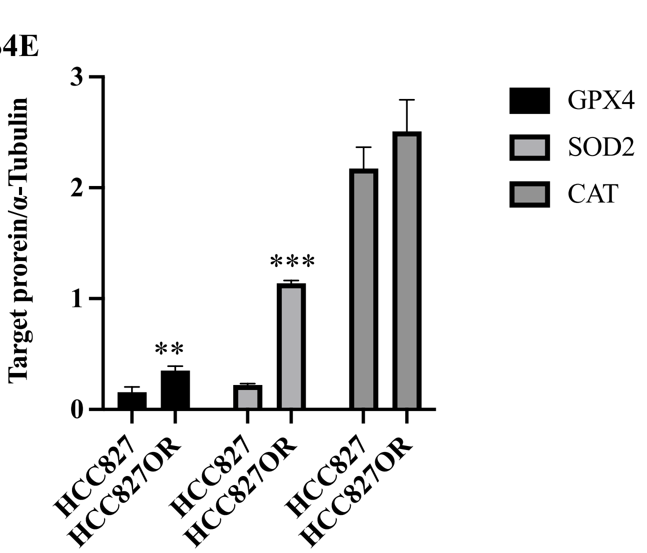

Supplement: Supplementary file 2 — Silver-stained gel, full western blots and quantification [file 41419_2026_9039_MOESM2_ESM.pdf]
